# Decoding the mechanism of proanthocyanidins in central analgesia: redox regulation and KCNK3 blockade

**DOI:** 10.1038/s12276-025-01412-5

**Published:** 2025-03-03

**Authors:** Junxiang Gu, Jian Wang, Hongwei Fan, Yi Wei, Yan Li, Chengwen Ma, Keke Xing, Pan Wang, Zhenyu Wu, Teng Wu, Xiaoyi Li, Luoying Zhang, Yunyun Han, Tao Chen, Jianqiang Qu, Xianxia Yan

**Affiliations:** 1 Department of Neurosurgery, the Second Affiliated Hospital of Xi’an Jiaotong University, Xi’an, China; 2 Department of Human Anatomy, Histology and Embryology and K.K. Leung Brain Research Centre, Fourth Military Medical University, Xi’an, China; 3 Department of Neurosurgery, Tangdu Hospital, Fourth Military Medical University, Xi’an, China; 4 Department of Pathophysiology, School of Basic Medicine and Tongji Medical College, Huazhong University of Science and Technology, Wuhan, China; 5 School of Medicine, Northwest University, Xi’an, China; 6 Shaanxi University of Chinese Medicine, Xianyang, China; 7 Key Laboratory of Molecular Biophysics of the Ministry of Education, College of Life Science and Technology, Huazhong University of Science and Technology, Wuhan, China; 8 Department of Neurobiology, School of Basic Medicine and Tongji Medical College, Huazhong University of Science and Technology, Wuhan, China

**Keywords:** Ion channels in the nervous system, Drug development

## Abstract

Neuropathic pain causes enduring physical discomfort and emotional distress. Conventional pharmacological treatments often provide restricted relief and may result in undesirable side effects, posing a substantial clinical challenge. Peripheral and spinal redox homeostasis plays an important role in pain processing and perception. However, the roles of oxidative stress and antioxidants in pain and analgesia on the cortical region during chronic pain remains obscure. Here we focus on the ventrolateral orbital cortex (VLO), a brain region associated with pain severity and involved in pain inhibition. Using a spared nerve injury mouse model, we observed the notable reactive oxygen species (ROS)-mediated suppression of the excitability of pyramidal cells (PYR^VLO^) in the VLO. Nasal application or microinjection of the natural antioxidants proanthocyanidins (PACs) to the VLO specifically increased the activity of PYR^VLO^ and induced a significant analgesic effect. Mechanistically, PACs activate PYR^VLO^ by inhibiting distinct potassium channels in different ways: (1) by scavenging ROS to reduce ROS-sensitive voltage-gated potassium currents and (2) by acting as a channel blocker through direct binding to the cap structure of KCNK3 to inhibit the leak potassium current (*I*_leak_). These results reveal the role of cortical oxidative stress in central hyperalgesia and elucidate the mechanism and potential translational significance of PACs in central analgesia. These findings suggest that the effects of PACs extend beyond their commonly assumed antioxidant or anti-inflammatory effects.

## Proanthocyanidins enhance pain relief in brain cortex

Neuropathic pain is a challenging condition that affects the nervous system, causing abnormal sensations and heightened sensitivity. Researchers explored the potential of proanthocyanidins, natural antioxidants found in plants, to alleviate neuropathic pain. The study involves experiments on mice with nerve injuries to mimic neuropathic pain. Researchers administered proanthocyanidins to the ventrolateral orbitofrontal cortex, a key brain region for pain regulation. They measured pain responses and examined brain activity using various techniques, including electrode recordings and molecular analysis. Results showed that proanthocyanidins decreased pain sensitivity in mice by lowering oxidative stress and modulating specific potassium channels that help control nerve cell activity. This dual mechanism—antioxidant effects and potassium channel regulation—highlights proanthocyanidins as an alternative to conventional pain treatments.

This summary was initially drafted using artificial intelligence, then revised and fact-checked by the author.

## Introduction

Neuropathic pain is a serious clinical condition that commonly arises from lesions or diseases affecting the peripheral or central nervous system. Patients with neuropathic pain typically experience abnormal sensations and frequently report heightened sensitivity to mechanical and thermal stimuli^1^. Although many aspects of neuropathic pain have been elucidated, unraveling its pathogenesis, and identifying effective treatments still pose formidable challenges. The currently widely used analgesic drugs, such as opioid receptor agonists and nonsteroidal anti-inflammatory drugs, often have suboptimal efficacy characterized by uncertain actions and side effects^2,3^. Therefore, the need to seek alternative analgesic agents has become urgent and imperative.

Redox homeostasis, the balance between oxidants (reactive oxygen species (ROS)) and antioxidants, plays an important role in pain processing and perception^4,5^. Excessive ROS levels in the spinal cord are considered to contribute to the initiation and persistence of neuropathic pain^6–8^. Intraperitoneal or intrathecal administration of ROS scavengers such as 4-hydroxy-2,2,6,6-tetramethylpiperidine-*N*-oxyl (Tempol) and PBN (α-phenyl-*N-tert-*butylnitrone) exerts potent analgesic effects on rodent models of various pain conditions^9–11^. However, pain-related oxidative stress and the possible analgesic effects of antioxidants in the supraspinal region remain to be determined.

In the context of neuropathic pain, cortical regions associated with pain sensitization, including the anterior cingulate cortex, insula cortex and primary somatosensory cortex, typically display increased activity^12–14^. Conversely, the ventrolateral orbitofrontal cortex (VLO), known for its role in descending pain inhibition through direct output to the ventrolateral periaqueductal gray, tends to show decreased activity^15,16^. A first-in-human study indicated that decreased neural activity in the orbitofrontal cortex can be used to predict high pain states in patients with refractory neuropathic pain^17^. In addition, optogenetic, chemogenetic or pharmacological activation of the VLO has analgesic effects on rodent models^15,18^. However, the redox regulation of pain-related brain regions, especially the pain inhibition region of the VLO, has not yet been elucidated.

Proanthocyanidins (PACs) are a group of polyphenolic compounds present in various plants. Unlike the synthetic antioxidants Tempol and PBN, PACs are natural antioxidants renowned for their potential health benefits. PACs are reported to alleviate acute and chronic pain. However, the current understanding of the analgesic effects of PACs is confined to peripheral tissue through their anti-inflammatory and antioxidant properties^19^. However, whether and how PACs can directly affect neuronal activity remain obscure. In our previous works, for the first time, we reported that the application of PACs in the spinal cord and insular cortex elicits analgesic effects^20,21^. Nevertheless, the mechanism underlying the antinociceptive action of PACs in the central nervous system is still largely unexplored.

In the present study, we aim to utilize PACs to investigate redox regulation at the cortical level during neuropathic pain and explore its possible analgesic effects on the VLO. We observed a decrease in the excitability of pyramidal cells (PYR^VLO^) and an increase in ROS levels in the VLO of spared nerve injury (SNI) model mice. The application of PACs decreased ROS production, enhanced the activity of PYR^VLO^ and induced a significant analgesic effect. Interestingly, PACs exerted their influence by inhibiting distinct potassium currents in different ways: one involved the suppression of ROS-sensitive voltage-gated potassium (Kv) currents through the scavenging of excess ROS, and the other involved the inhibition of the leak potassium current (*I*_leak_) by acting as a potential antagonist of KCNK3. Our work has, thereby, revealed the mechanism underlying the role of PACs in central analgesia and highlighted their potential value for clinical applications.

## Materials and methods

### Animals

Male C57BL/6 mice (6–8 weeks, 20–26 g) and glutamic acid decarboxylase 67-enhanced green fluorescent protein (GAD67–GFP) transgenic mice (JAX#007677, The Jackson Laboratory) were used for the experiments. The mice were housed in polyacrylic cages with food and water available ad libitum and maintained under pathogen-free conditions with a controlled temperature (24 ± 2 °C), humidity (50 ± 10%) and photoperiod (12–12 h light–dark cycle).

### Ethics

All animal procedures described in the present study were performed in accordance with protocols approved by the Animal Care Committee (IACUC-20210901) of the Fourth Military Medical University. All efforts were made to minimize animal suffering and the number of animals used.

### Drugs

PACs (oligomeric proanthocyanidins, UV ≥95%, CAS no. 4852-22-6) were purchased from Yuanye Biotechnology. Tempol (CAS no. 2226-96-2) was purchased from MedChemExpress. These reagents were dissolved in artificial cerebrospinal fluid (ACSF) to produce an original solution and were diluted to final concentrations of 50 μM (PACs) and 1 mM (Tempol) in ACSF for behavioral and electrophysiological experiments.

### Neuropathic pain model

The SNI model of neuropathic pain was established as described in our previous study^22^. Briefly, the mice were anesthetized by an intraperitoneal injection of 2% pentobarbital sodium (0.5 ml per 100 g). Using sterile technique, a small incision was made in the left thigh to expose the branches of the sciatic nerve. The tibial and common peroneal branches were ligated with 6–0 silk sutures and transected distal to the ligation, leaving the sural branch intact. Thereafter, the muscle and skin were sutured in two layers. For the sham surgery, the three branches of the sciatic nerve were exposed but not ligated or transected. A heating pad was used to maintain the animal’s body temperature during anesthesia and recovery.

### Brain stereotaxic procedures

#### Intracerebral guide cannula implantation

The mice were anesthetized and positioned in a stereotaxic apparatus (RWD Life Science). A small craniotomy was performed above the right VLO (coordinates: anterior–posterior (AP) +2.5 mm, mediolateral (ML) −1.1 mm and dorsoventral (DV) −2.5 mm). A single stainless steel guide cannula (no. 62004; outer diameter (OD) 0.41 mm, inner diameter (ID) 0.25 mm; RWD Life Science) was implanted 0.2 mm above the right VLO coordinates using a stereotactic holder. The cannula was fixed with two screws and dental acrylic cement (Super-Bond C and B). A stainless steel stylet plug (no. 62104, OD 0.2 mm, RWD Life Science) was inserted into the guide cannula.

#### In vivo electrode implantation

As previously described^23,24^, a 16-channel electrode array with a guide tube containing 16 electrodes (formvar-insulated nichrome wires) was implanted to target the right VLO (coordinates: AP +2.5 mm, ML −1.1 mm and DV −2.3 mm). The array was mounted to a microdrive to allow the advancement of the electrodes across subsequent testing days. Two silver ground wires were secured to the skull screws. A metal head bar, which was used to hold the mice firmly during subsequent recording, was also attached to the skull.

#### Intracerebral injection of AAV

The injection was performed as previously described^21^ to label excitatory (pyramidal) neurons in the VLO. Briefly, 300 nl of the viral suspension (rAAV-CAMKIIa-EGFP) was loaded into a glass micropipette (tip ID 10–20 μm) attached to a 1 μl Hamilton microsyringe. The micropipette was lowered into the VLO (coordinates: AP +2.5 mm, ML −1.1 mm and DV −2.5 mm), and the virus was injected via a microinjection pump (RWD Life Science) at a constant speed of 15 nl min^−1^. After the injection, the micropipette was left in place for an additional 20 min to minimize leakage.

The placement of the cannula and electrode and the injection site of the virus were verified post hoc by performing immunofluorescence staining.

### Drug administration

#### Intracerebral microinjection

After recovery from cannula implantation, the intracerebral microinjection was performed through an injector cannula (no. 62204; OD 0.21 mm, ID 0.11 mm; RWD Life Science) connected to a microsyringe driven by a microinfusion pump. The injector cannula was inserted into and protruded 0.5 mm beyond the guide cannula. A volume of 0.5 μl of PACs (50 μM) was injected at a rate of 0.1 μl min^−1^. The injector cannula was left in place for an additional 5 min to minimize leakage. During the microinjection, the mice were anesthetized with 2% isoflurane. The behavioral tests were conducted 30 min after PACs administration.

#### Intranasal delivery

The intranasal delivery of drugs to awake mice was performed according to a previously reported method^25^. A volume of 20 μl of PACs (50 μM) was loaded into a pipettor. After the mice were restrained, the tip of the filled pipettor was placed near the nostrils of each mouse at a 45° angle. The drug was slowly ejected to form a small droplet and the droplet was placed close enough to the mouse’s nostril to allow the mouse to inhale the droplet. If any droplets were snorted out, that drop was replaced. Behavioral tests were conducted at least 10 min after delivery.

### Pain behavior tests

#### Mechanical allodynia

The paw withdrawal mechanical threshold (PWMT) was measured via the simplified up and down *von Frey* test^26^. The mice were placed separately in inverted plexiglass boxes (7 cm × 7 cm × 10 cm) on an elevated mesh floor. A logarithmic series of eight calibrated *von Frey* filaments (Semmes Weinstein) from 0.008 to 2.0 g were applied to stimulate the plantar hindpaw. Positive responses included toe flinching or licking. A middle filament was first used, and then the next filament was selected according to the up–down rules until five presentations were completed. During the test, each grade of *von Frey* filament was bent and held for 3 s with an interval of 5 min.

#### Allodynia score

Dynamic mechanical allodynia was tested as previously described^27^. The mice were positioned as described for testing mechanical allodynia. The plantar hindpaw was stimulated with a paintbrush from heel to toe. A score of 0 corresponded to walking away or occasionally raising the foot. A score of 1 corresponded to raising the foot for more than 2 s or a single gentle retreat. A score of 2 corresponded to strongly raising the foot above the body level. A score of 3 corresponded to continuously shrinking or licking the foot. Each mouse was measured three times at 3 min intervals, and the average score of the measurements was calculated.

#### Thermal hyperalgesia

The paw withdrawal thermal latency (PWTL) was measured using the Hargreaves test with a plantar tenderness instrument (Ugo Basile), as described in a previous study^28^. The mice were placed individually in a plexiglass box mounted on the plate of the thermal stimulator. The radiation light spot was placed immediately below the hindpaw. The radiation intensity was adjusted to result in a latency of 8–15 s in the sham mice. The time between light onset and the foot lift was defined as the PWTL. Each test was repeated three times at 10 min intervals. A 20 s cutoff time was set to avoid skin damage.

#### CPP

PACs-mediated conditioned place preference (CPP) was assessed in a three-chamber box with two outer conditioning chambers connected by a neutral central compartment (Shanghai Vanbi Intelligent Technology). The two conditioning chambers have distinct visual and tactile cues with different walls (horizontal or vertical stripes) and floor textures (grid or hole). The PACs-mediated CPP protocol involved three phases^29^: the pretraining test at baseline (day 1), the training period (days 2–9) and the post-training test for PACs-mediated CPP (day 10). During the pretraining test, the mice were placed in the central compartment and allowed to explore both chambers freely for 30 min. The time spent in each chamber was recorded to assess any inherent preference. The side where the mice spent less time was assigned as the PACs-paired (conditioned) side, and the other side was identified as the ACSF-paired (unconditioned) side. During training days (days 2–9), the mice were confined to one chamber for 30 min following the intranasal administration of either PACs (50 μM in 20 μl) or an equal volume of ACSF. The mice were trained once a day and received PACs and ACSF on alternating days, with four training sessions. During the post-training test (day 10), the mice were placed in the middle compartment and allowed to explore both chambers freely for 30 min.

Video data were analyzed using a Pelco video processor and Tracking Master software connected to a computer. The mean time was calculated as the ratio of the time spent in the conditioned side to the total time spent in both chambers. The preference scores were calculated as the difference in time spent between the PACs-conditioned and unconditioned sides and reported as a percentage of the total time spent in both chambers.

### Single-unit recordings and spike sorting

Before the recording, the mice were habituated to be held firmly in the head-fixed setup in a soundproof chamber for at least 30 min a day for three consecutive days. Single-unit recordings in the VLO were performed with a NeuroLego System (Jiangsu Brain Medical Technology), bandpass filter of 0.3–6 kHz, sampling frequency of 30 kHz and amplitude threshold of 50 μV. On each recording day, a 30 min baseline recording was first performed, with a 20 min interval after drug delivery, followed by a 30 min drugged recording. After recording, the electrodes were moved down 60 μm steps to prepare for the next recording day.

Single-unit spike sorting was performed as previously described^22,30^. The data were analyzed offline using a MATLAB toolbox (MClust-v4.4). The waveforms with similar characteristics were manually defined into clusters. A cluster of waveforms was considered a single neuron if the ratio of its interspike interval under 2 ms was <1%, and the unit quality was quantified by the isolation distance (>20) and the L ratio (<0.1). If the spike time of any two units coincided via the cross-correlation comparison, these units were also defined as a single neuron. Three parameters, namely, the firing rate, trough-to-peak duration and half width, were used to differentiate putative excitatory pyramidal neurons (PYR^VLO^) and inhibitory interneurons (INT^VLO^) via an unsupervised clustering algorithm based on the *k*-means method. The trough-to-peak duration was particularly effective in separating PYR and INT: spikes with less than 430 μs were mainly classified as originating from INT^VLO^; otherwise, they were from PYR^VLO^.

### Whole-cell patch-clamp recording

The whole-cell patch-clamp recordings were performed as previously described^24^. Briefly, the mice were anesthetized with 3% isoflurane and decapitated. The whole brain was isolated and submerged in ice-cold (4 °C) and oxygenated (95% O_2_ and 5% CO_2_) ACSF (NaCl 124 mM, NaHCO_3_ 25 mM, KCl 2.5 mM, NaH_2_PO_4_ 1 mM, CaCl_2_ 2 mM, MgCl_2_ 1 mM and d-glucose 10 mM). Coronal slices (300 μm thick) containing the VLO were cut on a vibrating microtome (Leica VT 1200s, Heidelberger) and allowed to recover for 30 min at 37 °C and then at room temperature (25 °C) in oxygenated ACSF.

The slices were placed in a recording chamber and continuously perfused with oxygenated and heated (30 °C) ACSF at a rate of 5 ml min^−1^. mCherry-labeled PYR or GFP-labeled INT in the VLO were visualized under a fluorescence microscope (Olympus BX51WI). The recordings were performed in voltage-clamp or current-clamp mode using an Axon Multiclamp 700B amplifier (Molecular Devices). Clampex and Clampfit 10.2 software (Molecular Devices) were used to acquire and analyze the data. When the whole-cell configuration was achieved, the basic membrane properties and viability of the patched cells, including the resting membrane potential (RMP), rheobase (minimum current required to elicit an action potential), input resistance and spike number, were recorded. Rheobase was determined by current steps with 5 pA increments (30 ms). The membrane input resistance was calculated from hyperpolarizing voltage steps with −10 mV increments (400 ms) from a holding potential of −70 mV. The action potentials were induced by current steps from 0 to 300 pA with an increment of 25 pA (400 ms). The pipettes (5–7 MΩ) were filled with a recording solution composed of 124 mM K-gluconate, 5 mM NaCl, 1 mM MgCl_2_, 0.2 mM EGTA, 2 mM MgATP, 0.1 mM Na_3_GTP, 10 mM HEPES buffer and 10 mM phosphocreatine disodium (adjusted to pH 7.2 with KOH, 290 mOsm) to record the membrane properties. The initial access resistance was less than 35 MΩ and was changed to <15% throughout the experiment; otherwise, the result was discarded. Then, 0.2% biocytin (Sigma‒Aldrich) was introduced into the recording solution to identify the recorded neurons.

### Potassium current isolation

The potassium currents were isolated as previously reported^31,32^. The K^+^ currents from both Kv and subthreshold membrane currents were isolated by adding tetrodotoxin (1 μM) and CdCl_2_ (100 mM) into the ACSF to block the voltage-gated Na^+^ and Ca^2+^ currents.

The total Kv current was evoked by a protocol in which the holding potential was −70 mV, followed by conditioning hyperpolarization to −120 mV (200 ms) and then step voltages from −60 to 60 mV with 10 mV increments (800 ms). The Kv current subtype—the transient outward potassium current (Ito) and delayed outward rectifying potassium current (IKr)—were further determined by adding a prepulse to −40 mV before the step voltages to eliminate Ito, and the remaining currents recorded were identified as IKr. Ito was isolated by subtracting IKr from the total Kv currents.

Subthreshold membrane currents were recorded starting from a holding potential of −70 mV by applying voltage steps from −150 to −30 mV with an increment of −10 mV. I_leak_ was inhibited by perfusion with BaCl_2_ (5 mM), and the BaCl_2_-sensitive current was identified as I_leak_. The current amplitude was normalized to the cell capacitance to obtain the current density (pA/pF).

### Tonic GABA recording

Using a whole-cell patch-clamp recording configuration, the recording was conducted as described by Lee et al.^33^. The PYR^VLO^ were held at −70 mV, and a Cs-based internal solution was used with the following composition: 140 mM CsCl, 5 mM NaCl, 0.5 mM CaCl_2_, 10 mM HEPES, 5 mM EGTA, 2 mM MgATP, 0.5 mM Na_3_GTP and 2 mM QX-314 bromide (adjusted to pH 7.3 with CsOH, 290 mOsm). D-AP5 (50 μM) and CNQX (20 μM) were bath applied to pharmacologically isolate the inhibitory postsynaptic currents (IPSCs) and stabilize the baseline. The amplitude of the tonic γ-aminobutyric acid (GABA) current was measured by the baseline shift after the puff application of bicuculline (100 μM). The tonic current density was calculated by dividing the current amplitude by the membrane capacitance. The frequency and amplitude of spontaneous IPSCs before bicuculline administration were detected and measured using the Mini Analysis Program (Synaptosoft).

### Immunofluorescence staining

The mice were deeply anesthetized and perfused with 50 ml of 0.9% saline followed by 100 ml of 0.1 M PB containing 4% (w/v) paraformaldehyde. The brains were removed and gradient dehydrated with 30% sucrose for 24–48 h at 4 °C. Afterward, a series of 30-μm-thick sections containing the VLO were cut using a freezing microtome (Kryostat 1720; Leitz). The sections were collected in sequence, washed with 0.01 M PBS buffer and then rinsed with PBS containing 0.3% Triton X-100 and 1% normal goat serum for 0.5 h. For biocytin, glial fibrillary acidic protein (GFAP) and GABA immunofluorescence staining, the selected brain sections were incubated with antibiotin-Alexa Fluor 594 (1:1,000; S32356, Invitrogen), mouse anti-GFAP (1:4,000; MAB3402, MerckMillipore) and guinea pig anti-GABA (1:500; AB175, Sigma‒Aldrich, Germany) antibodies. All the antibodies were diluted in PBS containing 5% (v/v) normal donkey serum, 0.3% (v/v) Triton X-100, 0.05% (w/v) NaN_3_ and 0.25% (w/v) carrageenan (PBS–normal donkey serum, pH 7.4) overnight at 4 °C. Then, the sections were washed and incubated with the appropriate fluorophore-conjugated secondary antibody (1:200, Invitrogen, Thermo Fisher) for 4 h at room temperature. The fluorescence images were (directly observed) recorded using a laser scanning confocal microscope (FV1000, Olympus).

### Western blotting

The mice were killed after being deeply anesthetized, and the whole brains were quickly removed. The VLO was isolated and homogenized with an automatic rapid grinder (JXFSTRP-24L) in sodium dodecyl sulfate sample buffer. The samples were then heated at 100 °C for 10 min and loaded and electrophoresed on 10% sodium dodecyl sulfate‒polyacrylamide gels with standard Laemmli solutions (Bio-Rad Laboratories). The proteins were electroblotted onto a polyvinylidene difluoride membrane (Immobilon-P, Millipore). The membranes were incubated with the blocking solution for 1 h, followed by an overnight incubation with rabbit anti-GFAP (1:1000; ab68428; Abcam) and mouse anti-β-actin (1:5000; Sigma-Aldrich) primary antibodies. The bound primary antibodies were detected using a horseradish peroxidase-conjugated anti-rabbit (1:5000; ZB-2301, ZSGB-BIO) or anti-mouse (1:5000; ZB-2305, ZSGB-BIO) secondary antibody. Enhanced chemiluminescence (ECL) was used to visualize the bands, and the band densities were quantified using LabWorks Software (Ultraviolet Products).

### ROS detection

The ROS activity in the tissues was detected using a tissue ROS detection kit (dihydroethidium). A total of 50 mg of fresh brain tissue washed with PBS was added to 1 ml of homogenization buffer A and thoroughly homogenized using a glass homogenizer. After centrifugation at 4 °C (1000 *g*, 10 min), the supernatant was collected, and the pellet was discarded. Next, 190 μl of the supernatant and 10 μl of the dihydroethidium probe were added to a black 96-well plate and mixed thoroughly. The plate was incubated in the dark for 30 min at 37 °C, after which the fluorescence intensity of the samples was measured using a fluorescence microplate reader (excitation wavelength, 488 nm; emission wavelength, 610 nm). In addition, 50 μl of the supernatant was diluted 30 times with PBS, and 100 μl was used for protein quantification. Finally, the fluorescence intensity was divided by the amount of protein per milligram of tissue to represent the activity of the ROS produced in the tissue.

### In silico analysis of PACs-mediated ion channel activity

#### Selection of compounds

A thorough literature review revealed several compounds isolated from PACs. However, a monomeric compound isolated from PACs was selected for this study. The structures of PACs (Chem ID: 122173182) were downloaded from the PubChem database.

#### Preparation of ligands

Before performing molecular docking, the compounds were prepared by the Ligprep 3.3 wizard in Schrödinger Suite-Maestro v12.8 (ref. ^34^). Individual ligands were applied with the optimized potentials for liquid simulations (OPLS) force field, where three-dimensional geometries were created and proper bond orders were assigned. The ligands were neutralized at pH 7.0 ± 2.0 using Epik3.1 of the Schrödinger Suite. The lowest energy ring conformation of each ligand was selected for docking.

#### Preparation of the receptor and grid generation

The three-dimensional crystal structures of KCNA1 (PDB entry 1EXB), KCND2 (PDB entry 7E8E), KCNH1 (PDB entry 8EP1), KCNH5 (PDB entry 7YID), KCNK1 (PDB entry 7SK1), KCNK2 (PDB entry 6W83), KCNK3 (PDB entry 6RV4), KCNK4 (PDB entry 7LJB) and KCNK9 (PDB entry 3SMK) were downloaded in pdb format from the RCSB Protein Data Bank. The structures of the protein targets were prepared and refined using the Protein Preparation Wizard of Schrödinger Suite-Maestro v12.8. The charges and bond orders were assigned, and other necessary refinements were performed through standard protocols^35^. A properly computed receptor grid of arranged amino acid residues of proteins is needed, as each ligand has a specific binding site in the receptor. The grids were created, keeping the default parameters of a van der Waals scaling factor of 1.00 and a charge cutoff of 0.25 subjected to the OPLS force field. A cubic box was generated around the active site of the receptor with a measurement of 3 Å × 3 Å × 3 Å for docking experiments.

#### Glide standard precision ligand docking

Molecular docking was performed using Glide standard precision ligand docking^36^. The lowest Glide score for each ligand was considered the best-docked mode. Finally, the best-docked poses were further analyzed using Maestro v12.8 for two-dimensional and three-dimensional binding interactions with amino acid residues.

### The modified Hodgkin–Huxley model

The single-compartment neuron model was based on the Hodgkin‒Huxley (HH) model. The firing behavior of PYR^VLO^ was fit by extending the model through the incorporation of two additional types of potassium ion channel with a slow K^+^ current (${\bar{g}}_{\mathrm{M}}$) and delayed rectifying voltage-gated K^+^ current (${\bar{g}}_{{\mathrm{K}}_{{\mathrm{delayed}}}}$)

$${C}_{m}\frac{{{d}}{E}}{{{d}}{t}}=I-{\bar{g}}_{{{Na}}}{m}^{3}h\left(E-{E}_{{{Na}}}\right)-{\bar{g}}_{{{k}}}{n}^{4}\left(E-{E}_{{{K}}}\right)\,-\,{\bar{g}}_{{{L}}}\left(E-{E}_{{{L}}}\right)-{\bar{g}}_{{{k}}_{{{\mathrm{delayed}}}}}{{n}_{{{delayed}}}}^{4}\left(E-{E}_{{K}}\right){{\rm{\gamma }}}_{{{k}}_{{{{delayed}}}}}-{\bar{g}}_{{M}}p(E-{E}_{{K}})$$

$$\frac{{{d}}{m}}{{{{d}}t}}={\alpha }_{m}(E)(1-m)-{\beta }_{m}(E)m,\quad\frac{{{{d}}h}}{{{{d}}t}}={\alpha }_{h}\left(E\right)\left(1-h\right)-{\beta }_{h}\left(E\right)h,\quad\frac{{{{d}}n}}{{{{d}}t}}={\alpha }_{n}\left(E\right)\left(1-n\right)-{\beta }_{n}\left(E\right)n$$

$$\frac{{\mathrm{d}}{n}_{{\mathrm{delayed}}}}{{{\mathrm{d}}t}}={\alpha }_{{n}_{{\mathrm{delayed}}}}\left(E\right)\left(1-{n}_{{\mathrm{delayed}}}\right)-{\beta }_{{n}_{{\mathrm{delayed}}}}\left(E\right){n}_{{\mathrm{delayed}}},\quad\frac{{{\mathrm{d}}p}}{{{\mathrm{d}}t}}=\frac{{p}_{{{\infty }}}\left(E\right)-p\left(E\right)}{{\tau }_{p}\left(E\right)}$$

$${\alpha }_{m}\left(E\right)=\frac{-0.32\left(E-{E}_{{T}}-13\right)}{{{e}}^{\frac{-(E-{E}_{{T}}-13)}{4}}-1},\quad{\beta }_{m}\left(E\right)=\frac{0.28\left(E-{E}_{{T}}-40\right)}{{{e}}^{\frac{-(E-{E}_{{T}}-40)}{5}}-1},\quad{\alpha }_{h}(E)=-0.128{{e}}^{\frac{-(E-{E}_{{T}}-17)}{18}},\quad{\beta }_{h}(E)=\frac{4}{1+{{e}}^{\frac{-(E-{E}_{{T}}-40)}{5}}},$$

$${\alpha }_{n}(E)={\gamma }_{\alpha_n}\frac{-0.032(E-{E}_{{T}}-20)}{{{e}}^{\frac{-(E-{E}_{{T}}-20)}{5}}-1},\quad{\beta }_{n}(E)={\gamma }_{\beta_n}0.5{{e}}^{\frac{-(E-{E}_{{T}}-20)}{40}}$$

$${\alpha }_{{n}_{{{delayed}}}}(E)={\gamma }_{{\alpha }_{{n}_{{{delayed}}}}}\frac{-0.032(E-{E}_{{T}}-\Delta {V}_{{{k}}_{{{delayed}}}})}{{{e}}^{\frac{-(E-{E}_{{T}}-\Delta {V}_{{{k}}_{{{delayed}}}})}{5}}-1},\quad{\beta }_{{n}_{{{delayed}}}}(E)={\gamma }_{{\beta }_{{n}_{{{delayed}}}}}0.5{{e}}^{\frac{-(E-{E}_{{T}}-\Delta {V}_{{{k}}_{{{delayed}}}})}{20}}$$

$${p}_{{{\infty }}}(E)=\frac{1}{1+{{e}}^{\frac{-(E+33)}{10}}},\quad{\tau }_{p}(E)=\frac{{\tau }_{max }}{3.3{{e}}^{\frac{-(E+33)}{20}}+{{e}}^{\frac{-(E+33)}{20}}},$$

where *E*(*t*) is the membrane potential; ${C}_{m}$ is the cell capacitance; ${E}_{\mathrm{L}}$, ${E}_{{\mathrm{Na}}}$ and ${E}_{\mathrm{K}}$ are the leak, sodium and potassium reversal potentials; *m*(*E*, *t*) is the sodium channel activation; *h*(*E*, *t*) is the sodium channel activation; *n*(*E*, *t*) is the potassium channel activation; ${n}_{{\mathrm{delayed}}}(E, t)$ is the delayed potassium channel activation; *p* is the slow potassium channel activation^37^; ${\bar{g}}_{\mathrm{L}}$ is the leak conductance; ${\bar{g}}_{{\mathrm{Na}}}$ is the maximum conductance of the voltage-gated sodium channel; and ${\bar{g}}_{\mathrm{k}}$, ${\bar{g}}_{{\mathrm{k}}_{\rm{delayed}}}$ and $\quad{\bar{g}}_{M}$ are the maximum conductances of the Kv channel, the delayed rectifying potassium channel and the slow potassium channel, respectively. The values used in the HH models are listed in Table 1. Modulation of the activation, deactivation and voltage sensitivity of the delayed potassium channel and slow potassium channel is determined by introducing the parameters ${\gamma }_{\alpha_{n_{{delayed}}}}$, ${\gamma }_{\beta_{n_{{delayed}}}}$, ${{\rm{\gamma }}}_{{\mathrm{k}}_{{delayed}}}$ and $\Delta {V}_{{\mathrm{k}}_{{delayed}}}$, respectively^38^. The first two scale the rate constants (${\alpha }_{{n}_{{{delayed}}}}$ and ${\beta }_{{n}_{{{delayed}}}}$). The parameter $\Delta {V}_{{\mathrm{k}}_{{delayed}}}$ simultaneously shifts the voltage sensitivity of the rate constants ${\alpha }_{{n}_{{\mathrm{delayed}}}}(E)$ and ${\beta }_{{n}_{{{delayed}}}}(E)$.

**Table 1:** Parameters of the HH model used to simulate the firing patterns under sham, SNI and PACs treatment conditions.

	${\bar{g}}_{\mathrm{L}}({\mathrm{mS}}\,{{\mathrm{cm}}}^{-2})$	${\bar{g}}_{{\mathrm{{K}_{{delayed}}}}}\,({\mathrm{mS}}\,{{\mathrm{cm}}}^{-2})$	${\bar{g}}_{\mathrm{M}}({\mathrm{mS}}\,{{\mathrm{cm}}}^{-2})$
Sham	0.115	8.0	0.01
Sham + PACs	0.070	8.0	0.01
SNI	0.115	9.5	0.3
SNI + PACs (Kv)	0.115	8.0	0.2
SNI + PACs (leak)	0.070	9.5	0.3
SNI + PACs (leak + Kv)	0.070	8.0	0.2

The rest of the model parameters are the same for all the conditions. ${C}_{m}=0.7\,\upmu {\mathrm{F}}\,{{\mathrm{cm}}}^{-2}$, $S=4\times 1{0}^{-5}\,{\rm{c}}{{\rm{m}}}^{2}$, ${E}_{\mathrm{L}}=-70.0\,{\mathrm{mV}}$, ${E}_{{\mathrm{Na}}}=40.0\,{\mathrm{mV}}$, ${E}_{\mathrm{K}}=-70.0\,{\mathrm{mV}}$, ${E}_{\mathrm{T}}=-52.0\,{\mathrm{mV}}$, ${\bar{g}}_{{\mathrm{Na}}}=50\,{\mathrm{mS}}\,{{\mathrm{cm}}}^{-2}$, ${\bar{g}}_{K}=1.28\,{\mathrm{mS}}\,{{\mathrm{cm}}}^{-2}$, ${\gamma }_{\alpha_n}=1$, ${\gamma }_{\beta_n}=0.30$, ${\gamma }_{\alpha_{n_{\mathrm{delayed}}}}=0.110$, ${\gamma }_{{\beta }_{{n}_{{\mathrm{delayed}}}}}=0.006$, ${{\rm{\gamma }}}_{{\mathrm{k}}_{\mathrm{delayed}}}=1$, $\Delta {V}_{{\mathrm{k}}_{\rm{delayed}}}=22.0\,{\mathrm{mV}}$ and ${\tau }_{\max }=1$

### Statistics

Statistical analyses and data visualization were performed using GraphPad Prism 10 (GraphPad Software). All data shown in the column and line graphs are presented as the mean ± standard error of the mean, unless mentioned otherwise. All data from the samples were tested for the normality of distribution using the Shapiro‒Wilk test before being assessed using two-tailed two-sample *t-*tests, two-tailed paired *t-*tests, two-tailed Mann‒Whitney tests, two-tailed Wilcoxon matched-pairs signed rank analysis or two-way analysis of variance followed by a post hoc comparison using the Bonferroni correction. Statistical significance is indicated as */^#^*P* < 0.05, **/^##^*P* < 0.01; ***P < 0.001 and ****P < 0.0001.

## Results

### VLO microinjection or intranasal administration of PACs induces analgesia in SNI mice

The SNI model is widely acknowledged for its utility in studying neuropathic pain^39^. Compared with the sham mice, ROS levels in the VLO of SNI mice were significantly elevated on days 3, 7 and 21 postsurgery. The intranasal administration of PACs significantly reduced the ROS levels in the VLO of SNI mice at each of these time points (Supplementary Fig. 1a, b). The pain behaviors were also assessed in animals at different time points after SNI (Supplementary Fig. 1a). SNI mice exhibited significant decreases in mechanical (PWMT) and thermal (PWTL) pain thresholds, as well as a substantial increase in the allodynia score on days 3, 7 and 21 postsurgery, indicating the presence of sustained allodynia and hyperalgesia (Supplementary Fig. 1c–e). Upon the intranasal administration of PACs (50 μM, 20 μl), we found that PACs significantly increased the PWMT and PWTL and reduced the allodynia score in SNI mice at each of these time points (Supplementary Fig. 1c–e). Furthermore, this analgesic effect of PACs appeared to last for more than 4 h (Supplementary Fig. 1f, g).

**Fig. 1:**
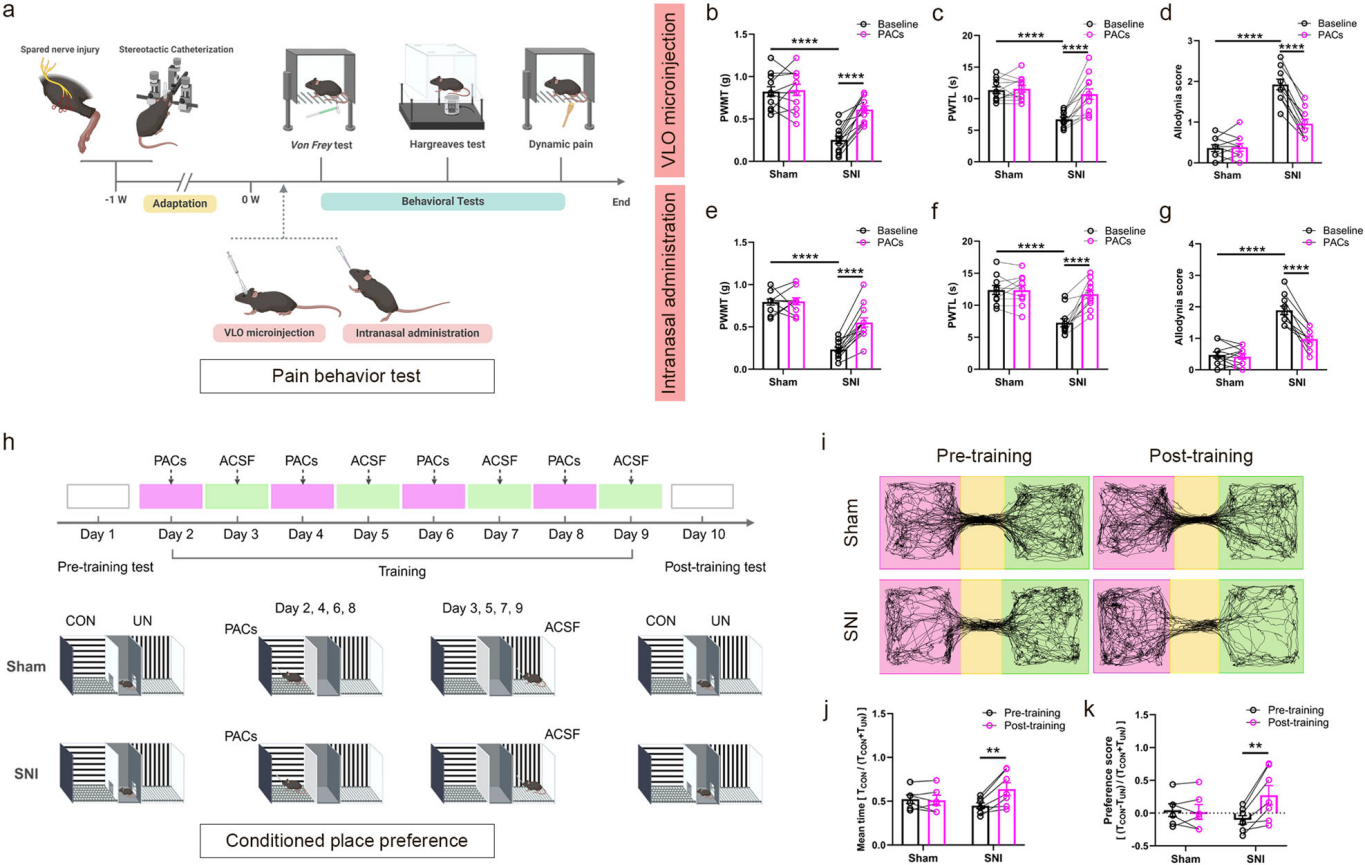
VLO microinjection or intranasal administration of PACs induces analgesia and CPP in SNI mice. **a** A schematic of SNI, drug delivery and experimental timeline. **b**–**g** The baseline data of VLO microinjection and intranasal administration were combined, significant reductions in PWMT (sham: *n* = 23, SNI: *n* = 25; *P* < 0.0001) and PWTL (sham: *n* = 22, SNI: *n* = 24; *P* < 0.0001) and a substantial increase in allodynia score (sham: *n* = 21, SNI: *n* = 21, *P* < 0.0001) were observed in the SNI mice. The microinjection of PACs into the VLO significantly increases the PWMT (SNI-baseline versus SNI-PACs, *n* = 12 mice, *P* < 0.0001) (**b**), the PWTL (SNI-baseline versus SNI-PACs, *n* = 13 mice, *P* < 0.0001) (**c**) and decreases the dynamic mechanical allodynia score (SNI-baseline versus SNI-PACs, *n* = 10 mice, *P* < 0.0001) (**d**) of SNI mice. The intranasal administration of PACs markedly increases the PWMT (SNI-baseline versus SNI-PACs, *n* = 13 mice, *P* < 0.0001) (**e**), the PWTL (SNI-baseline versus SNI-PACs, *n* = 11 mice, *P* < 0.0001) (**f**) and decreases the dynamic mechanical allodynia score (SNI-baseline versus SNI-PACs, *n* = 11 mice, *P* < 0.0001) (**g**) in SNI mice. **h** A timeline and diagram of PACs-mediated CPP. CON, conditioned side (paired with PACs); UN, unconditioned side (paired with ACSF). **i** The representative traces of mice in the sham group and SNI group in the pretraining test on day 1 (left) and post-training test on day 10 (right). **j** The summary data for the mean time spent in the conditioned side during the pretraining test and post-training test (SNI: pretraining versus post-training, *n* = 7, *P* = 0.0073). **k** The summary data for the preference scores during the pretraining test and post-training test (SNI: pretraining versus post-training, *n* = 7, *P* = 0.0073). *T*_CON_, time spent in the conditioned side; *T*_UN_, time spent in the unconditioned side. **P < 0.01 and ****P < 0.0001. Source data in Dataset 1.

A cannula was also implanted into the right VLO for the local microinjection of PACs. On day 7 after surgery, the microinjection of PACs (50 μM, 0.5 μl) significantly increased the PWMT and PWTL and reduced the dynamic allodynia score of SNI mice but had no effect on sham mice (Fig. 1a–d). These effects of locally delivered PACs were comparable to those observed with the intranasal administration of PACs (50 μM, 20 μl) (Fig. 1a, e–g and Supplementary Fig. 1), indicating the significant analgesic efficacy of PACs in treating neuropathic pain, regardless of whether they were delivered locally into the VLO or broadly via intranasal administration.

CPP is a valuable tool for exploring how animals perceive and respond to spontaneous pain relief as they develop a preference for environments associated with pain relief^40^. In this study, we performed a three-chamber CPP paradigm, pairing one chamber with PACs treatment (PACs-conditioned side) and the other with a control ACSF solution (unconditioned side) based on the mice’s pretraining preference (Fig. 1h). During the training phase, the mice were alternately exposed to the PAC- and ACSF-paired chambers. Following training, SNI mice spent significantly more time in and showed a greater preference for the PACs-conditioned side (Fig. 1i–k). In contrast, the sham mice displayed no change in their preference before or after training. These results suggest that PACs treatment has potent spontaneous pain-relieving effects.

### PACs specifically increase the firing rate of PYR^VLO^ in SNI mice in vivo

**Fig. 2:**
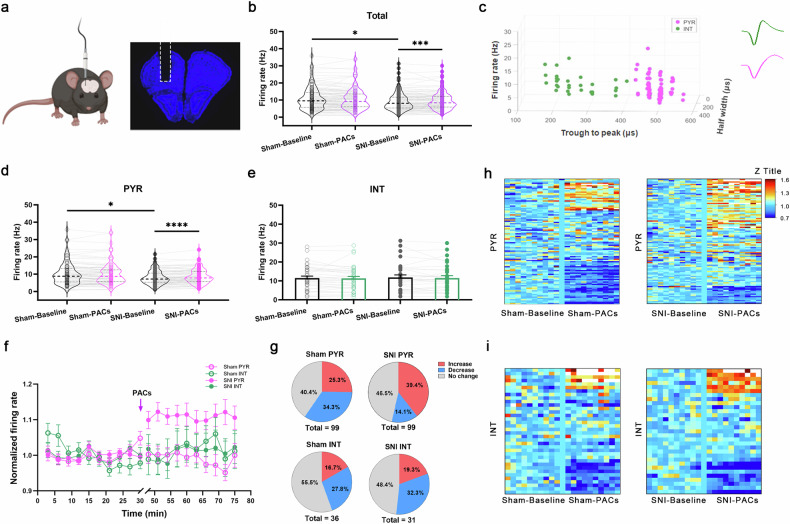
Intranasal application of PACs increases the firing rate of PYR^VLO^ in SNI mice. **a** Schematic of in vivo multichannel recordings (left) and a representative slice showing an electrode implantation trace in the VLO (right). **b** Total firing rate of VLO neurons in sham (*n* = 135 neurons) and SNI (*n* = 130 neurons) mice before (sham-baseline versus SNI-baseline, *P* = 0.0426) and after (SNI-baseline versus SNI-PACs, *P* = 0.0002) PACs application. **c** VLO neurons are classified as PYR^VLO^ (purple dots) and INT^VLO^ (green dots) using the *k*-means cluster-separation algorithm based on their firing properties. **d** Scatter plots showing the firing rate of PYR^VLO^ in sham (*n* = 99 neurons) and SNI (*n* = 99 neurons) mice (sham-baseline versus SNI-saseline, *P* = 0.0271) before and after PACs application (SNI-baseline *versus* SNI-PACs, *P* < 0.0001). The lines in the scatter plots represent the medians. **e** Scatter plots showing the firing rate of INT^VLO^ in sham (*n* = 36 neurons) and SNI (*n* = 31 neurons) mice. **f** Pooled data showing the normalized firing rates of PYR^VLO^ and INT^VLO^ in sham and SNI mice before and after PACs application. The error bars represent the mean ± standard error of the mean. The purple arrow indicates PACs administration. **g** Pie charts showing the proportions of PYR^VLO^ and INT^VLO^ with increased, decreased and unchanged firing rates in the sham and SNI mice after PACs application. **h**, **i** *Z*-score heat maps depicting the changes in the firing rates of PYR^VLO^ and INT^VLO^ in sham and SNI mice after PACs application. *P < 0.05, ***P < 0.001 and ****P < 0.0001. Source data in Dataset 1.

To clarify the possible excitability changes of the VLO neurons after SNI and to elucidate the impact of PACs on those neurons, we implanted a 16-channel electrode in the VLO and conducted in vivo real-time single-unit recordings of neuronal discharges before and after the administration of PACs (Fig. 2a). A total of 130 neurons were isolated in the VLO of SNI mice based on their well-matched spiking patterns before and after PACs administration, with an average firing rate of 8.80 ± 0.46 Hz. In the sham mice, a total of 135 neurons were well isolated, with an average firing rate of 10.29 ± 0.56 Hz. The firing rate of neurons in SNI mice was significantly lower than that in sham mice (Fig. 2b), indicating that the VLO neuronal activity is inhibited in SNI mice. We then intranasally administered PACs (50 μM, 20 μl) and found that PACs selectively and significantly increased the firing rate of VLO neurons in SNI mice but not in sham mice (Fig. 2b).

To distinguish whether PACs application specifically affected the neuronal firing of pyramidal cells (PYR^VLO^) or interneurons (INT^VLO^) within the VLO, we used three parameters (trough-to-peak duration, half-width and firing rate) at baseline for neuronal classification. We identified that spikes with shorter trough-to-peak duration (<430 μs) were mainly originated from the INT^VLO^ (Fig. 2c). Among the 130 neurons recorded in SNI mice, 99 were classified as PYR^VLO^ and 31 were classified as INT^VLO^. In sham mice, 99 PYR^VLO^ and 36 INT^VLO^ were sorted among the 135 neurons. We found that the firing rate of PYR^VLO^ was significantly decreased in SNI mice (Fig. 2d), and this decrease was reversed by the intranasal administration of PACs (Fig. 2d, f). Similarly, the application of PACs increased the firing rate of a larger portion (39.4%) of PYR^VLO^ in SNI mice than it did in sham mice (25.3%). Moreover, PACs decreased the firing rate of a smaller portion (14.1%) of PYR^VLO^ in SNI mice than in sham mice (34.3%) (Fig. 2g, h). In contrast, the firing rate of INT^VLO^ remained unchanged between SNI and sham mice and was generally unaffected by PACs application (Fig. 2e–g, i).

### PACs increase the excitability of PYR^VLO^ in both sham and SNI mice in vitro

**Fig. 3:**
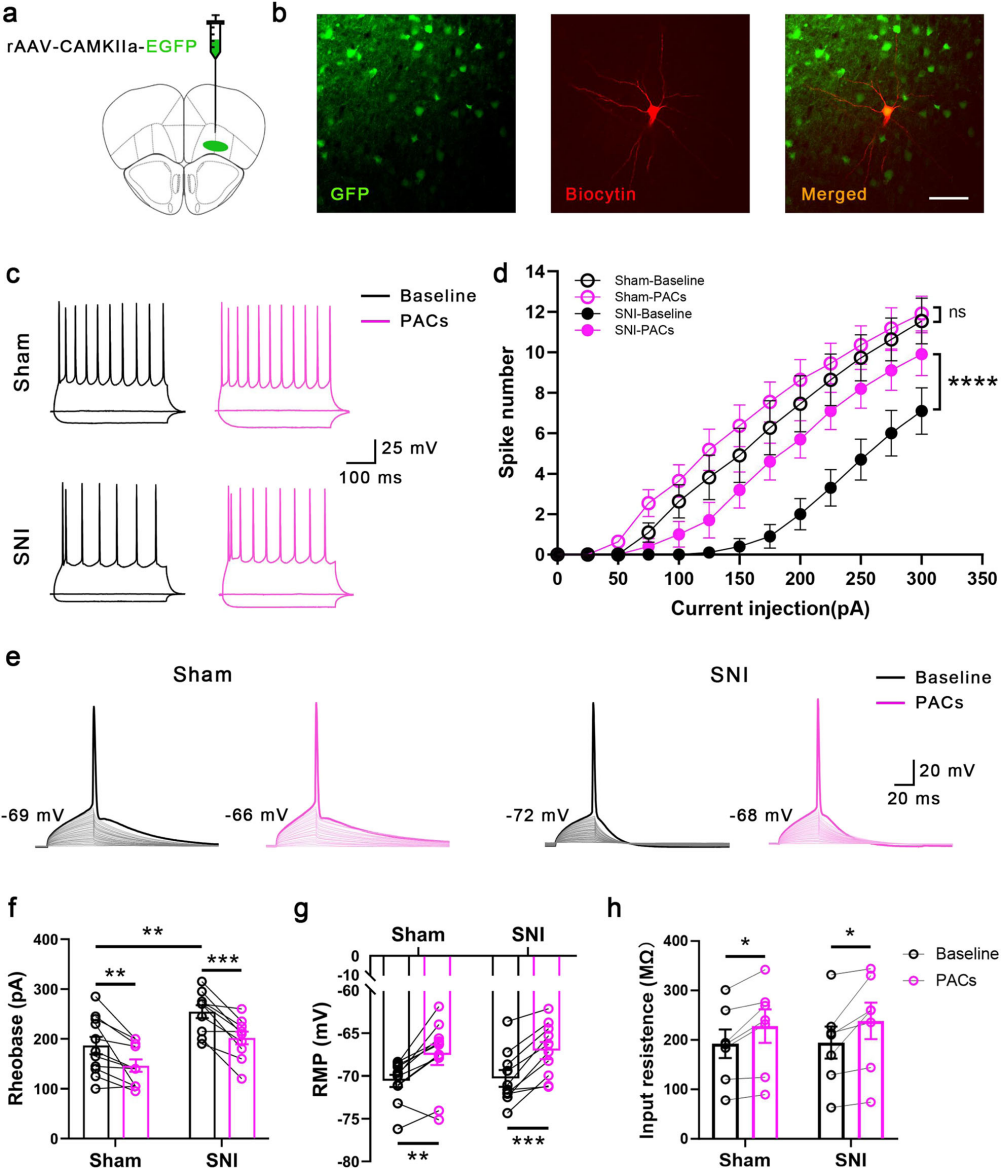
PACs potentiate the excitation of PYR^VLO^ in both sham and SNI mice and specifically increase the number of evoked spikes in SNI mice. **a** A schematic of the virus injection. **b** Images showing GFP^+^ pyramidal neurons (left), one whole-cell patched neuron labeled with biocytin and stained with TRITC (middle) and the merged image (right). **c** Representative traces of spikes of PYR^VLO^ elicited by 300 pA current stimulation before and during PAC application in sham and SNI mice. **d** Summary data for the spikes evoked by step depolarizing currents in sham and SNI mice (sham-baseline (*n* = 11) versus SNI-baseline (*n* = 10), *p* < 0.0001) before and during PACs application (SNI-baseline versus SNI-PACs, *n* = 10, *P* < 0.0001). **e** Representative traces of the first action potential generated by step current stimulation in PYR^VLO^ before and during PACs application in sham and SNI mice. **f** Rheobase changes in PYR^VLO^ during the application of PACs in sham and SNI mice (sham-baseline (*n* = 11) versus SNI-baseline (*n* = 10), *P* = 0.0060; sham-baseline versus sham-PACs, *n* = 11, *P* = 0.0047; SNI-baseline versus SNI-PACs, *n* = 10, *P* = 0.0005). **g** RMP changes in PYR^VLO^ during the application of PACs in sham and SNI mice (sham-baseline versus sham-PACs, *n* = 11, *P* = 0.0027; SNI-baseline versus SNI-PACs, *n* = 10, *P* = 0.0003). **h** Input resistance changes in PYR^VLO^ during the application of PACs in sham and SNI mice (sham-baseline versus sham-PACs, *n* = 7, *P* = 0.0125; SNI-baseline versus SNI-PACs, *n* = 7, *P* = 0.0091). *P < 0.05, **P < 0.01, ***P < 0.001, ****P < 0.0001; n.s., not significant. Source data in Dataset 1.

The in vivo recording results indicate that PACs specifically increase the excitability of PYR^VLO^ in SNI mice. We further investigated the effects of PACs on the electrophysiological properties of PYR^VLO^ and INT^VLO^ by performing in vitro whole-cell patch-clamp recordings. After the injection of AAV2/9-CaMKIIα-GFP into the VLO, PYR^VLO^ were recognized as GFP-labeled CaMKIIα-positive cells (Fig. 3a, b). Consistent with the results of the in vivo recordings, the number of spikes evoked by step depolarizing currents injected into PYR^VLO^ was significantly decreased in SNI mice. Bath application of PACs (50 μM) increased the number of spikes in SNI mice but not in sham mice (Fig. 3c, d). We then analyzed the membrane property parameters of PYR^VLO^. Compared with those in sham mice, the rheobase of PYR^VLO^ in SNI mice was increased, whereas the RMP and input resistance (*R*_in_) remained unchanged (Fig. 3e–h). However, bath application of PACs not only decreased the rheobase but also elevated the RMP and increased the *R*_in_ of PYR^VLO^ in both sham and SNI mice (Fig. 3e–h). Further analysis of the action potential waveform parameters in PYR^VLO^ revealed that the decreased decay time (90–10%) in SNI mice was also reversed by PACs application (Supplementary Fig. 2).

INT^VLO^ were visualized and recorded using GAD67–GFP transgenic mice (Supplementary Fig. 3a,b). Unlike those in PYR^VLO^ neurons, SNI treatment did not alter the evoked spiking or passive membrane properties of INT^VLO^ when compared with those of the sham mice. Moreover, the administration of PACs had a minimal effect on the INT^VLO^ in both the sham and SNI mice, with the only notable difference being a minor increase in the RMP of the sham mice (Supplementary Fig. 3c–h).

### PACs excite PYR^VLO^ by inhibiting distinct potassium currents

Compared with sham mice, SNI mice presented a decreased firing rate (Fig. 2d) and an increased rheobase (Fig. 3f) of PYR^VLO^, along with an increased rise time, maximum decay slope, decreased decay time and half-width of the action potential (Supplementary Fig. 2). These results strongly suggest that Kv channels, which regulate the firing, threshold and repolarization of action potential^41^, are involved in the inhibition of PYR^VLO^ activity in SNI mice. PACs could counteract this inhibition by modulating Kv channels.

**Fig. 4:**
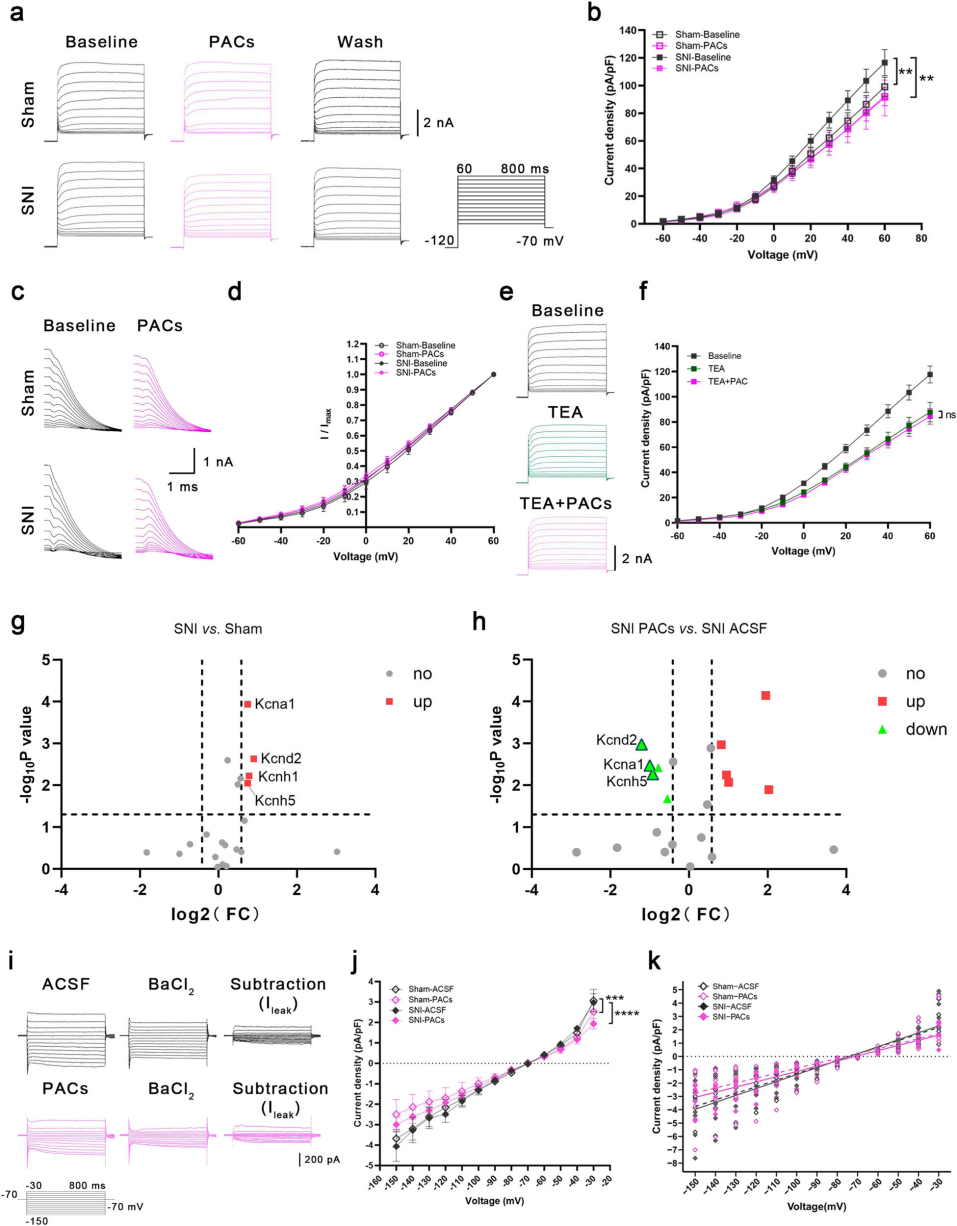
PACs specifically inhibit the increased Kv currents in SNI mice, but broadly inhibit *I*_leak_ in both sham and SNI mice. **a** the representative traces of total Kv currents before and during the application of PACs and wash out in sham and SNI mice. **b** Current density‒voltage curves for total Kv currents in sham and SNI mice treated with and without PACs. The current density of Kv channels is increased in SNI mice (sham-baseline (*n* = 7) versus SNI-baseline (*n* = 8), *P* = 0.0011). PACs specifically inhibits the increased Kv currents in SNI mice (SNI-baseline versus SNI-PACs, *n* = 8, *P* = 0.0010). **c** Representative traces of the tail current of Kv channels before and during the application of PACs in sham and SNI mice. **d** The normalized tail currents (*I*/*I*_max_) of Kv channels. The voltage dependence of the steady-state activation of Kv channels does not significantly change during the application of PACs. **e** Representative traces of Kv currents during the application of TEA and the coapplication of TEA and PACs in SNI mice. **f** The current density‒voltage curves for Kv currents during the application of TEA and the coapplication of TEA and PACs in SNI mice. TEA blocks the inhibition of Kv currents caused by PACs in SNI mice (TEA versus TEA + PACs, *n* = 7, *P* > 0.9999). **g** Volcano plots showing the upregulated (red squares) Kv channels in SNI mice (fold change (FC) >1.5). Sham, *n* = 3 mice; SNI, *n* = 3 mice. **h** Volcano plots showing the changes in the expression of Kv channels after the application of PACs in SNI mice (FC >1.5). ACSF, *n* = 3 mice; PACs, *n* = 3 mice. **i** The representative traces of *I*_leak_ in PYR^VLO^ with and without the application of PACs in SNI mice. **j** The current density‒voltage curves for *I*_leak_ in sham and SNI mice with and without the application of PACs. **k** Current density‒voltage scatter plots and their linear fits are applied as a function for estimating *I*_leak_ in sham and SNI mice with and without the application of PACs by comparing the slopes (sham-ACSF (*n* = 7) versus SNI-ACSF (*n* = 8), *P* = 0.4428; sham-ACSF (*n* = 7) versus sham-PACs (*n* = 8), *P* = 0.0004; SNI-ACSF (*n* = 8) versus SNI-PACs (*n* = 9), *P* < 0.0001). *P < 0.05, **P < 0.01; n.s., not significant. Source data in Dataset 1.

We validated this hypothesis by isolating the whole-cell potassium currents of PYR^VLO^ in the presence of tetrodotoxin (1 μM) and CdCl_2_ (100 μM) to block voltage-gated Na^+^ and Ca^2+^ currents. The current‒voltage (*I‒V)* curve of total potassium currents showed a strong voltage dependence and outward rectification characteristics (Supplementary Fig. 4a,b), indicating the predominance of Kv currents in depolarization-activated potassium currents. Consistent with our hypothesis, SNI mice presented an increase in the current density of total Kv currents (Fig. 4a, b), which was reversed by PACs application without affecting the steady-state activation of Kv channels (Fig. 4a–d). When we used tetraethylammonium (TEA, 10 mM) to block the delayed Ikr, the predominant component of the Kv currents in PYR^VLO^ (Supplementary Fig. 4c, d), the PACs no longer exerted inhibitory effects (Fig. 4e, f). These findings indicate that PACs suppress the increase in Kv currents, specifically the TEA-sensitive Ikr, of PRY^VLO^ in SNI mice without altering the kinetics of Kv channels. We subsequently used a PCR array to examine the expression of Kv channels in the VLO and observed the notable upregulation of kcna1, kcnd2, kcnh1 and kcnh5 in SNI mice (Fig. 4g), among which PACs downregulated the expression of *kcna1*, *kcnd2* and *kcnh5* (Fig. 4h).

The RMP and *R*_in_ of PYR^VLO^ did not differ between sham and SNI mice, suggesting that the SNI treatment had a minimal effect on the passive membrane properties of PYR^VLO^. Nevertheless, PACs depolarized the RMP and increased the *R*_in_ of PYR^VLO^ in sham and SNI mice, indicating their potential effect on the inhibition of leak potassium channels (K2P) and/or inward rectifying potassium channels, as both channel types contribute to establishing the RMP and regulating membrane resistance^42,43^. However, the distinct outwardly rectifying nature of the *I‒V* curve for PYR^VLO^ precluded a further examination of the impact of PACs on inward rectifying potassium channels (Supplementary Fig. 4a,b). Consequently, the alterations in RMP and *R*_in_ induced by PACs might be attributed to their effects on K2P in PYR^VLO^. We isolated the leak potassium current (*I*_leak_) to investigate this possibility further and found that it remained unchanged between sham and SNI mice. However, PACs inhibited *I*_leak_ in both groups (Fig. 4i–k).

### PACs inhibit the augmented Kv currents of PYR^VLO^ in SNI mice by scavenging ROS

**Fig. 5:**
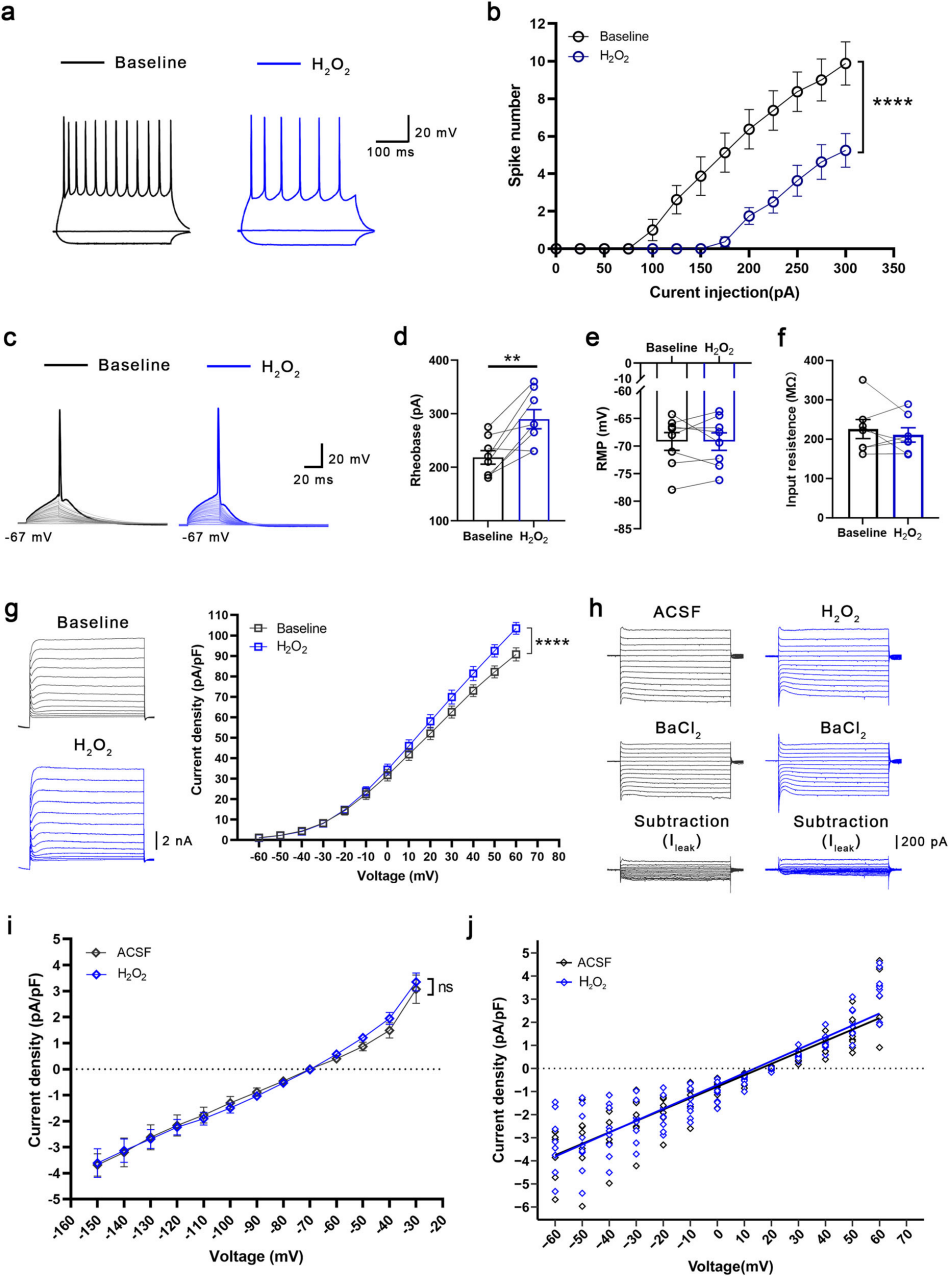
Administration of H_2_O_2_ to the VLO of sham mice mimics the changes in spikes and Kv currents in PYR^VLO^ after SNI. **a** Representative traces of the spikes of PYR^VLO^ elicited by a 300 pA current stimulation before and during the application of H_2_O_2_ in sham mice. **b** The summary data for the spikes evoked by step depolarizing currents in sham mice before and during the application of H_2_O_2_ (baseline versus H_2_O_2_, *n* = 8, *P* < 0.0001). **c** The representative traces of the first action potential generated by step current stimulation in PYR^VLO^ before and during the application of H_2_O_2_. **d** Rheobase of PYR^VLO^ before and during the application of H_2_O_2_ (baseline versus H_2_O_2_, *n* = 8, *P* = 0.0075). **e** RMP of PYR^VLO^ before and during the application of H_2_O_2_, *n* = 8. **f** The input resistance of PYR^VLO^ before and during the application of H_2_O_2_, *n* = 7. **g** Representative traces of Kv currents in PYR^VLO^ before and during H_2_O_2_ application (left); H_2_O_2_ significantly increased the current density of Kv channels (baseline versus H_2_O_2_, *n* = 7, *P* < 0.0001) (right). **h** Representative traces of *I*_leak_ in PYR^VLO^ with and without H_2_O_2_ application. **i** Current density‒voltage curves for *I*_leak_ with and without the application of H_2_O_2_. **j** Current density‒voltage scatter plots and their linear fits are applied as a function for estimating *I*_leak_ with and without the application of H_2_O_2_ by comparing the slopes (ACSF (*n* = 7) versus H_2_O_2_ (*n* = 9), *P* = 0.3430). *P < 0.05, **P < 0.01, ****P < 0.0001; n.s., not significant. Source data in Dataset 1.

We were interested in exploring how PACs influence the Kv and K2P currents in the VLO. ROS have been implicated in the development and maintenance of neuropathic pain^44^ and are known to regulate potassium channels in several physiological and pathological processes^45–47^. Given the robust antioxidant properties of PACs, we hypothesize that SNI treatment might induce oxidation in the VLO, thereby increasing the activity of potassium channels, especially Kv channels, and subsequently suppressing the spiking of PYR^VLO^. PACs could counteract this oxidative modulation by scavenging excess ROS. We tested this hypothesis by first ascertaining that the SNI treatment notably increased ROS levels in the VLO, which were then significantly reduced upon PACs administration (Supplementary Fig. 1b). After H_2_O_2_ (2 mM), the most prevalent cellular oxidant, was bath applied to the VLO slices of the sham mice, H_2_O_2_ significantly decreased the excitability of PYR^VLO^, as evidenced by a decreased number of evoked spikes and an increased rheobase, without altering RMP or *R*_in_ (Fig. 5a–f). Furthermore, H_2_O_2_ increased the current density of Kv channels in PYR^VLO^, leaving the *I*_leak_ unaffected (Fig. 5g–j). These observations closely resemble the electrophysiological shifts induced by SNI in PYR^VLO^ neurons (Figs. 3 and 4).

**Fig. 6:**
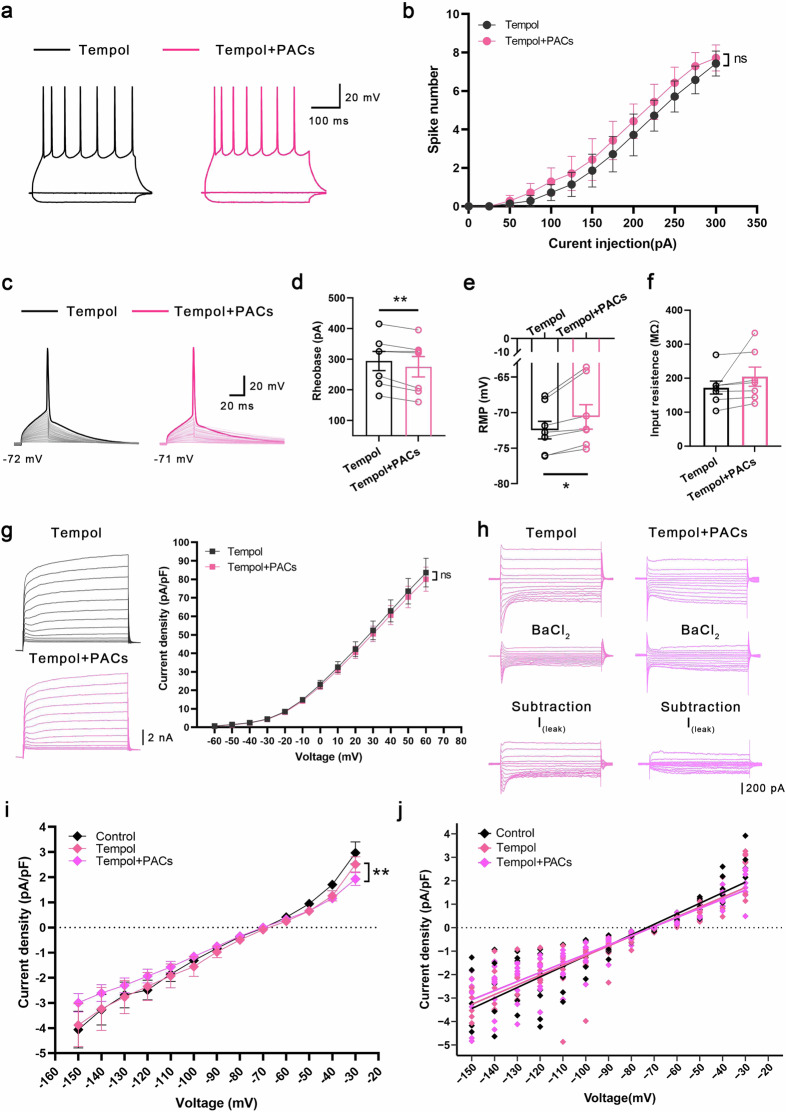
Tempol blocks the effects of PACs on spikes and Kv currents but not on *I*_leak_, in PYR^VLO^ in SNI mice. **a** Representative traces of the spikes of PYR^VLO^ elicited by a 300 pA current stimulation. **b** The summary data for the PYR^VLO^ spikes evoked by step depolarizing currents during the preapplication of Tempol and coapplication of Tempol and PACs, *n* = 7. **c** Representative traces of the first action potential generated by step current stimulation in PYR^VLO^ with the application of Tempol and coapplication of Tempol and PACs. **d** Rheobase of PYR^VLO^ during the application of Tempol and the coapplication of Tempol and PACs (*n* = 7, *P* = 0.0096). **e** RMP of PYR^VLO^ during the application of Tempol and coapplication of Tempol and PACs (*n* = 7, *P* = 0.0142). **f** Input resistance of PYR^VLO^ during the application of Tempol and coapplication of Tempol and PACs (*n* = 7, *P* = 0.1904). **g** Left: representative traces of Kv currents in PYR^VLO^ with the application of Tempol and the coapplication of Tempol and PACs. Right: PACs no longer affect the current density of Kv channels with the preapplication of Tempol, *n* = 9. **h** Representative traces of *I*_leak_ in PYR^VLO^ with the application of Tempol and PACs. **i** Current density‒voltage curves for *I*_leak_ with or without the application of Tempol and PACs. **j** Current density‒voltage scatter plots and their linear fits are applied as a function for estimating *I*_leak_ with or without the application of Tempol and PACs by comparing the slopes (control (*n* = 8) versus Tempol (*n* = 8), *P* = 0.2189; control (*n* = 8) versus PACs (*n* = 9), *P* = 0.0002; Tempol (*n* = 8) versus PACs (*n* = 9), *P* = 0.0086). *P < 0.05, **P < 0.01; n.s., not significant. Source data in Dataset 1.

We then tested whether the effects of PACs on Kv currents were mediated by scavenging of ROS. To this end, we employed Tempol, a membrane-permeable superoxide dismutase mimetic, to assess its potential to block the effects of PACs on SNI mice. Our findings revealed that, following Tempol administration, PACs no longer influenced the spiking activity of PYR^VLO^ but still decreased the rheobase and elevated the RMP (Fig. 6a–f). As anticipated, Tempol completely blocked the inhibitory effect of PACs on Kv currents but failed to block their inhibitory effect on *I*_leak_ (Fig. 6g–j). These results suggest that PACs can reverse the augmented Kv currents after SNI via their antioxidant effects, whereas their inhibition of *I*_leak_ might be independent of this redox regulation. Consistently, the analgesic effect of PACs was more potent and prolonged than that of Tempol, further indicating that the analgesic effect of PACs is not solely mediated by antioxidant mechanisms (Supplementary Fig. 1f, g).

### PACs inhibit the leak potassium current in PYR^VLO^ by directly binding with the cap structure of KCNK3

**Fig. 7:**
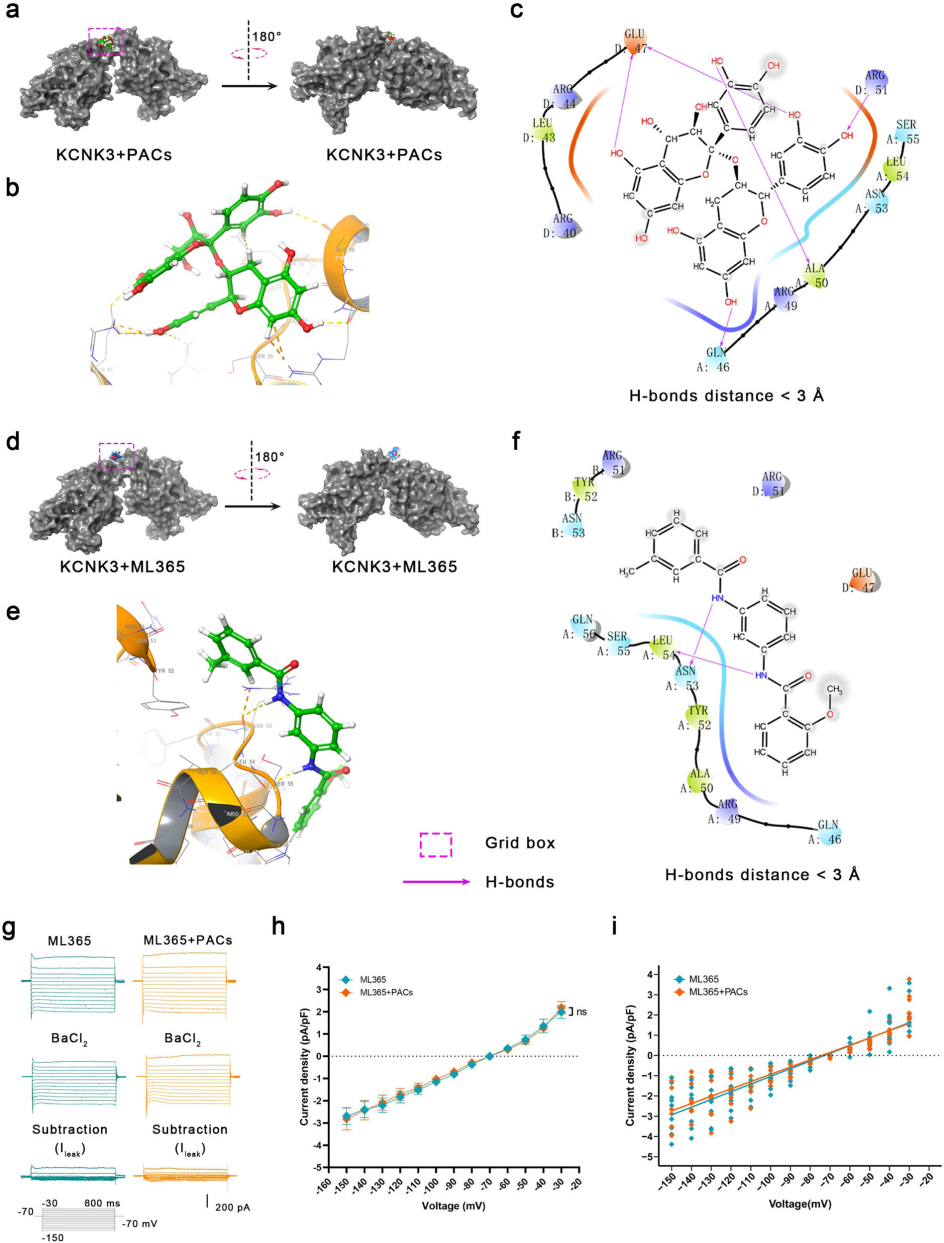
PACs inhibit the leak potassium current (I_leak_) in PYR^VLO^ by directly combining with the cap structure of KCNK3. **a**–**c** Docking mode between PACs and KCNK3. Overlay (**a**) and two-dimensional (**b**) interactions showing the docking active pocket highlighted in the grid box. Close-up views (**c**) showing the hydrogen bonds in two-dimensional interactions. **d**–**f** Docking mode between ML365 and KCNK3. Overlay (**d**) and two-dimensional (**e**) interactions showing the docking active pocket highlighted in the grid box. Close-up views (**f**) showing the hydrogen bonds in two-dimensional interactions. **g** Representative traces of *I*_leak_ in PYR^VLO^ during the application of ML365 and coapplication of ML365 and PACs in sham mice. **h** Current density‒voltage curves for *I*_leak_ in sham mice during the application of ML365 and the coapplication of ML365 and PACs. **i** Current density‒voltage scatter plots and their linear fits are applied as a function for estimating *I*_leak_ upon the application of ML365 and coapplication of ML365 and PACs by comparing the slopes (ML365 (*n* = 9) versus ML365 + PACs (*n* = 9), *P* = 0.999). ***P < 0.001, ****P < 0.0001; n.s., not significant. Source data in Dataset 1.

The PACs used in this study are oligomers of flavan-3-ol monomers. Multiple hydroxyl groups and aromatic ring structures enable their interactions with proteins through intermolecular forces such as hydrogen bonding, hydrophobic interactions, and van der Waals forces, which induce alterations in the protein conformation and impact protein function^48,49^. PACs broadly inhibited the *I*_leak_ in both sham and SNI mice, which may be due to a potential direct effect on R2P. We then performed molecular docking using Schrödinger Maestro to investigate the binding interactions of PACs with K2P expressed in the central nervous system. Notably, the docking results revealed direct interactions between PACs and KCNK3, KCNK2 and KCNK9. The binding sites on KCNK2 and KCNK9 are located in the transmembrane and intracellular domains, respectively (Supplementary Fig. 5a–f), with minimal effects on channel permeability to K^+^ (ref. ^50^). Specifically, PACs bound to the cap structure of KCNK3 (Fig. 7a). Within the active pocket of KCNK3, which contains amino acid residues from the 46th to the 56th positions, PACs form a single hydrogen bond with Gln46, Ala50 and Arg51 and two hydrogen bonds with Glu47 (Fig. 7b, c). The cap structure contributes to K^+^ channel pore formation and is a validated binding target for a highly selective antagonist (ML365) of KCNK3 (ref. ^51^). The binding site analysis of PAC-KCNK3 indicated that PACs occupy an active pocket in a manner similar to that of ML365 (Fig. 7d). ML365 forms hydrogen bonds with Leu53 and Asn54 of KCNK3 (Fig. 7e,f). The PAC-KCNK3 binding, which involves more hydrogen bonds, might be tighter. More importantly, in the presence of ML365 (5 μM), the application of PACs did not result in further inhibition of *I*_leak_ (Fig. 7g–i). These findings suggest that the inhibitory effect of PACs on the *I*_leak_ of PYR^VLO^ in both sham and SNI mice might be due to their potential role as an antagonist of KCNK3.

Similarly, a molecular docking platform (Schrödinger) was used to predict the possible binding of PACs with the downregulated Kv channels indicated in the PCR array (Fig. 4h). However, we found that PACs have no direct binding site in any of these channels (Supplementary Fig. 5g).

### The HH model reveals that PACs rescue the hypoexcitability of PYR^VLO^ via the inhibition of both Kv and K2P channels

**Fig. 8:**
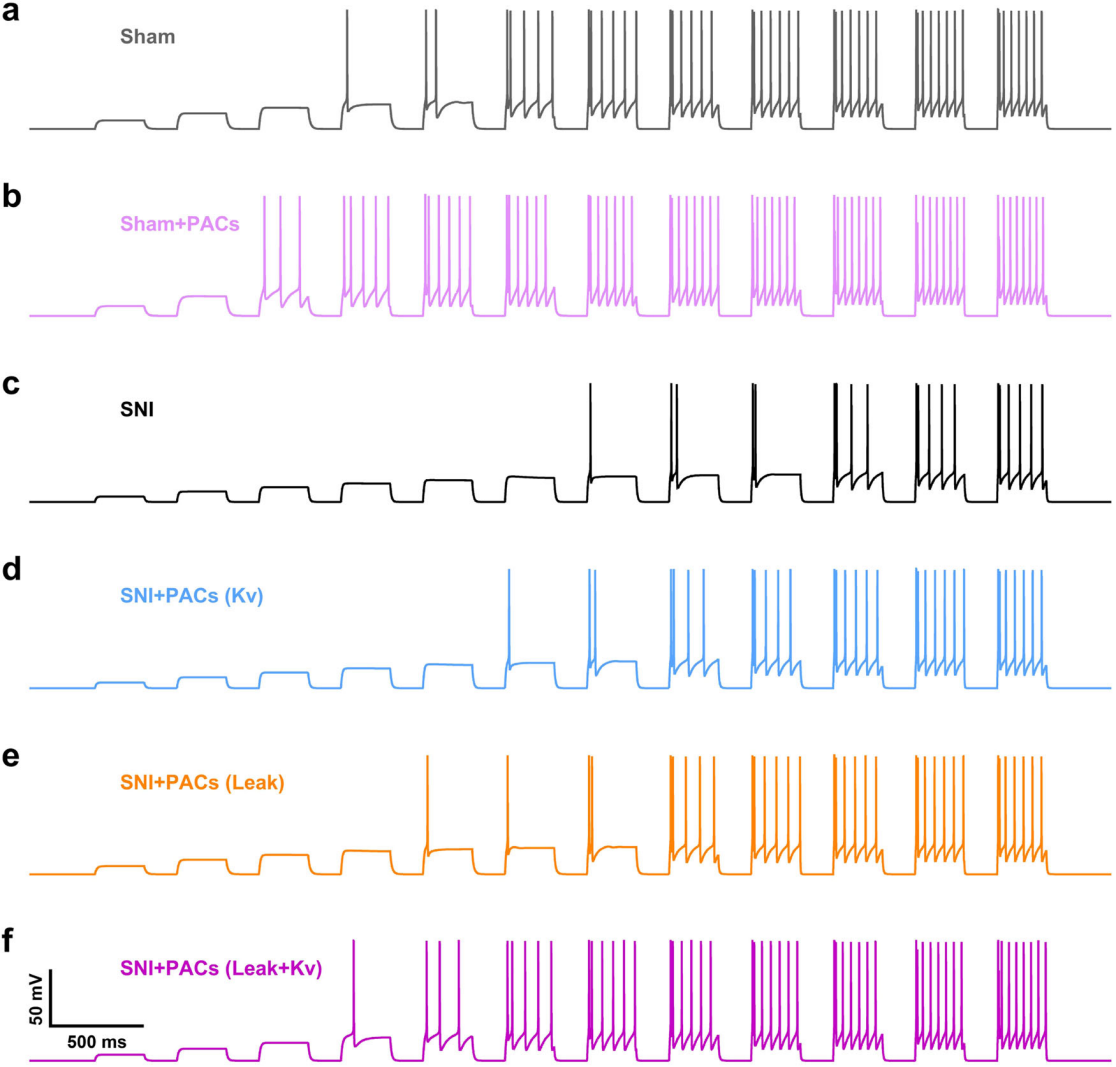
Suppression of Kv and K2P by PACs both contribute to the rescue of PYR^VLO^ excitability in SNI. **a** Baseline voltage response of PYR^VLO^ in the Sham group. **b** PACs block the leak current and slightly increased excitability in the sham group. **c** An increase in Kv currents induced by SNI results in reduced excitability. **d**, **e** The blocking effect on Kv currents (**d**) and leak currents (**e**) independently increases the firing response upon current injection in the SNI group. **f** Application of PACs, by blocking both types of current, rescues the deficit in excitability induced by SNI. The voltage response of PYR^VLO^ was simulated with the modified HH model upon the injection of step current pulses with a duration of 300 ms and interval of 200 ms with an increment of 25 pA per step. The parameters for the HH model are listed in Table 1.

Finally, we investigated how the regulation of voltage-gated K^+^ and leak channels resulted in the observed changes in the excitability of PYR^VLO^. We simulated the spike response of PYR^VLO^ upon current injection using the modified single-compartment Hodgkin–Huxley (HH) model^37,38^. The conductance of Kv and leak currents were modulated to mimic the effects of SNI and PACs. PACs were able to suppress K2P channels (KCNK3) in both sham and SNI mice (Figs. 4 and 7). A reduction in leak (${\bar{g}}_{\mathrm{L}}$) conductance slightly increased the action potential in sham mice (Fig. 8a, b). When the maximal total K^+^ conductance (${\bar{g}}_{\mathrm{M}}$ and ${\bar{g}}_{{\mathrm{{K}_{{delayed}}}}}$) was increased to mimic the SNI condition, as observed in the experiment (Fig. 8c), the excitability of SNI neurons was evidently reduced, as the rheobase needed to generate the action potential was increased and the number of action potentials induced by the large step current (300 pA) was also reduced (Fig. 8c). PACs suppressed both the Kv and K2P channels in SNI mice (Figs. 4 and 7). A reduction in either K^+^ (${\bar{g}}_{\mathrm{M}}$ and ${\bar{g}}_{{\mathrm{{K}_{{delayed}}}}}$) or leak (${\bar{g}}_{\mathrm{L}}$) conductance was sufficient to increase excitability in SNI mice; however, neither approach was capable of fully restoring excitability. This result was evidenced by the simulation results that the rheobase was higher than that observed in the sham group and that the maximum spikes upon 300 pA current injections remained lower (Fig. 8d, e). When PACs blocked Kv and K2P simultaneously, the dual effect more efficiently rescued the hypoexcitability induced by SNI. This property was observed by the simulation of the firing pattern upon the current injection of SNI neurons with the dual effects of PACs, which exhibited similar excitability to that of the sham group (Fig. 8f). Therefore, consistent with our in vitro whole-cell patch-clamp recordings, the effects of PACs on blocking Kv and K2P channels were both crucial for restoring the excitability that was impaired by SNI. The multitarget effects worked in concert to ensure the reliable analgesic effect of the PACs.

### The effects of PACs may not be achieved through the inhibition of astrocyte activation

ROS play a pivotal role in driving reactive gliosis in the brain. Studies have shown that reactive astrocytes aberrantly synthesize GABA and H_2_O_2_ (refs. ^52,53^) and that H_2_O_2_ scavenging can reduce reactive astrocyte activation and reduce astrocytic GABA-mediated tonic inhibition in the hippocampus of an Alzheimer’s disease mouse model^53^. In the spinal dorsal horn of a neuropathic pain model, reactive astrocytes and excessive GABA release have also been observed^54^. Therefore, it is interesting to investigate whether the analgesic effects of PACs are also mediated by inhibiting astrocyte activation and reducing astrocytic GABA-mediated inhibition.

Our immunofluorescence staining revealed an increase in the number of GFAP^+^ cells in the VLO of SNI mice, with some of these cells colocalized with GABA, suggesting the activation of astrocytes and astrocytic GABA-mediated tonic inhibition in the VLO following SNI (Supplementary Fig. 6a). Western blot analysis further confirmed elevated GFAP expression in the VLO of SNI mice, and the administration of PACs had the potential to reduce its expression (Supplementary Fig. 6b, c). However, patch-clamp recordings did not reveal significant differences in the frequency or amplitude of spontaneous IPSCs between the sham and SNI groups, and the application of PACs did not affect spontaneous IPSCs either (Supplementary Fig. 6d–f). We also observed a bicuculline (GABA_A_ receptor antagonist)-sensitive tonic GABAergic current (a shift in the membrane potential) in the patched PYR^VLO^. However, no significant difference in the amplitude of the tonic GABAergic current (averaging 8.45–13.74 pA) was observed among all the groups (Supplementary Fig. 6d, g, h). These findings suggest that although astrocyte activation occurs in the VLO after SNI, the phasic and tonic GABA-mediated inhibition of PYR^VLO^ may not be substantially changed. Moreover, PACs may not have a significant effect on astrocytic GABA-mediated tonic inhibition.

## Discussion

In the present study, we observe elevated levels of ROS within the VLO and the specific inhibition of PYR^VLO^ with increased Kv currents in mice with neuropathic pain. PACs exert a substantial analgesic effect by increasing the excitability of PYR^VLO^ through the inhibition of the ROS-sensitive Kv currents and the KCNK3-mediated *I*_leak_ in distinct ways. These findings provide insights into the central analgesic effect of PACs and potential therapeutic interventions targeting central oxidative stress and potassium channels.

### VLO inhibition during chronic neuropathic pain

Consistent with clinical electrophysiological findings^17^, we revealed that the firing rate of neurons in the VLO decreased in SNI mice, characterized by a reduction in both the firing rate and the decreased excitability of PYR^VLO^, whereas those of the INT^VLO^ remained unchanged. These findings align with those reported by Sheng et al.^55^, who observed significant decreases in both the spontaneous firing rate and the intrinsic excitability of PYR^VLO^ in a mouse model of trigeminal neuralgia induced by chronic constriction injury of the infraorbital nerve. Since PYR^VLO^ have been reported to directly activate glutamatergic neurons in the ventrolateral periaqueductal gray and, thus, increase the activity of the descending pain modulatory system^15^, which was also confirmed in our previous studies (unpublished data), the reduced firing rate of PYR^VLO^ should lead to the suppression of pain inhibition. Our study further revealed that the Kv currents in PYR^VLO^ increased after SNI, accompanied by the upregulation of certain Kv channels, which elucidates the ion channel mechanism underlying the inhibition of PYR^VLO^ during chronic pain.

Research on pain sensitization mediated by oxidative stress has focused primarily on the spinal cord, with a limited exploration of its effects on the pain-processing region of the cortex. Our study shows elevated ROS levels and the subsequent ROS-mediated inhibition of pyramidal cells in the VLO following chronic pain. Leveraging the proximity of the VLO to the cribiform plate, we intranasally administered the membrane-permeable antioxidant PACs to modulate VLO activity. Our findings revealed that PACs significantly reduced ROS levels in the VLO, resulting in analgesia. These findings provide valuable insights into the intricate interplay between oxidative stress and pain processing, paving the way for innovative therapeutic approaches for managing chronic pain by precisely targeting oxidative stress pathways in the cortex.

### Redox modulation of Kv channels in pain hypersensitivity and analgesia

Numerous studies have confirmed the downregulation of Kv channels in neurons within the dorsal root ganglia, spinal dorsal horn and somatosensory cortex during chronic pain. This downregulation often leads to increased neuronal excitability and subsequent pain hypersensitivity^56,57^. Conversely, in the VLO, we observed a notable upregulation of specific Kv subtypes, including Kv1.1 (KCNA1), Kv4.2 (KCND2), Kv10.1 (KCNH1) and Kv10.2 (KCNH5), with no downregulation of Kv channels after SNI. Electrophysiological studies further revealed an increase in Kv currents. Since PYR^VLO^ participates in pain inhibition circuits, increased Kv currents lead to the inhibition of PYR^VLO^, thereby enhancing nociception. More importantly, our results indicate that oxidation leads to increased activity of K^+^ channels. Previous research has shown that ROS regulate Kv channels through various mechanisms, including influencing their gene transcription and directly affecting channel proteins, auxiliary subunits or membrane components^47^. These interactions often involve modifications of specific residues, such as cysteine, methionine, tyrosine or histidine. Alterations in cellular ROS levels also disturb the redox balance, affecting channel function by modulating redox couples such as Fe^2+^/Fe^3+^ and GSH-reduction/GSH-oxidation^45,47^. These findings, which are consistent with our data, suggest that the elevated ROS levels in the VLO after SNI oxidatively modulate Kv channels, resulting in an increase in Kv currents. The increased currents, in turn, inhibit the activity of PYR^VLO^ and induce pain hypersensitivity.

PACs reduce ROS levels in the VLO, suppress augmented Kv currents, increase the excitability of PYR and exert analgesic effects. As natural antioxidants, these beneficial effects of PACs are probably achieved by reversing the ROS-induced oxidative modifications of Kv channels and potentially modulating various intracellular signaling pathways, such as the MAPK, Akt/mTOR and Nrf2 pathways, which may affect secondary messengers and thereby regulate Kv channel function^58^. After SNI, the expression of certain Kv subtypes was consistently increased. However, following the administration of PACs, most of the upregulated Kv genes (*kcna1*, *kcnd2* and *kcnh5*) were downregulated.

Interestingly, PACs induce similar analgesic effects but operate through distinct mechanisms under comparable chronic pain conditions. In our previous works^20,21^, PACs reduced the excitability of PYR in the insular cortex and spinal dorsal horn. Conversely, in the present study, they increased the excitability of PYR in the VLO. Whether the differential involvement of Kv channels accounts for this seemingly contradictory effect remains uncertain. Nevertheless, our PCR array findings indicate that PACs either downregulate or upregulate different Kv subtypes (Fig. 4h). These distinct channels might be differentially expressed in neurons across various regions^59,60^. Hence, the influence of PACs on neurons in different brain areas could be associated with their varying impacts on multiple Kv channels.

### PACs, potential blocker of KCNK3

In addition to the inhibition of Kv currents through redox regulation, PACs suppressed the *I*_leak_ of PYR^VLO^ in both sham and SNI mice. The molecular docking analysis indicated that PACs bind to the cap structure of KCNK3 channels, suggesting their direct antagonist effects. Notably, this cap structure is also the target of ML365, a commercialized antagonist of KCNK3. The cap structure functions as an archway at the channel pore entrance, creating a bifurcated pathway for K^+^ flux through the channel under physiological conditions. Therefore, PACs are likely to be potential blockers of KCNK3, and this study provides a report of a direct interaction between PACs and KCNK3. However, although these findings are supported by experimental evidence showing the inhibition of *I*_leak_ and predicted binding sites, further investigations are needed to elucidate the role of PACs as potential inhibitors of the KCNK3 channel using molecular pharmacological and/or structural biology methods. The effects of PACs on blocking Kv and leak currents might both contribute to their analgesic role. We used a single-compartment neuron model to estimate how each contributes to the regulation of PYR^VLO^ excitability under both sham and SNI conditions. PACs decreased the maximal K^+^ conductance (${\bar{g}}_{\mathrm{M}}$ and ${\bar{g}}_{{\mathrm{{K}_{{delayed}}}}}$) and increased the excitability of PYR^VLO^ in SNI animals. In addition, PACs blocked the leak conductance in both sham and SNI animals but increased excitability only in PYR^VLO^ of SNI mice, probably because PYR^VLO^ became more sensitive to the regulation of leaky conductance when Kv currents were upregulated by SNI. This property makes PACs more promising candidate analgesics than other general anti-ROS reagents because the additional blocking effect on leak channels does not interfere with the physiological function of PYR^VLO^ but plays an important role in the analgesic effect under chronic pain conditions.

We confirm that the regulation of oxidation in the VLO is important for neuronal activity and the process of neuropathic pain. PACs induce analgesic effects by enhancing the activity of PYR^VLO^ via the suppression of ROS-sensitive Kv currents and KCNK3-mediated *I*_leak_. These findings provide new insights into the oxidative mechanism underlying pain and suggest potential clinical applications of PACs in the treatment of chronic pain.

## Supplementary Information

Supplementary Information

Dataset 1

## Data Availability

The data supporting the results of this study are available within the manuscript and Supplementary Information. The source data and detailed statistical analysis for each figure are provided in the Dataset file.
