# Supplementary material for: Decoding the mechanism of proanthocyanidins in central analgesia: redox regulation and KCNK3 blockade
Source: Exp Mol Med. 2025 Mar 3;57(3):567–83. doi: 10.1038/s12276-025-01412-5 (PMC11958645; doi:10.1038/s12276-025-01412-5)
Supplement: Supplementary file 1 — Supplementary Information [file 12276_2025_1412_MOESM1_ESM.pdf]

Supplementary information

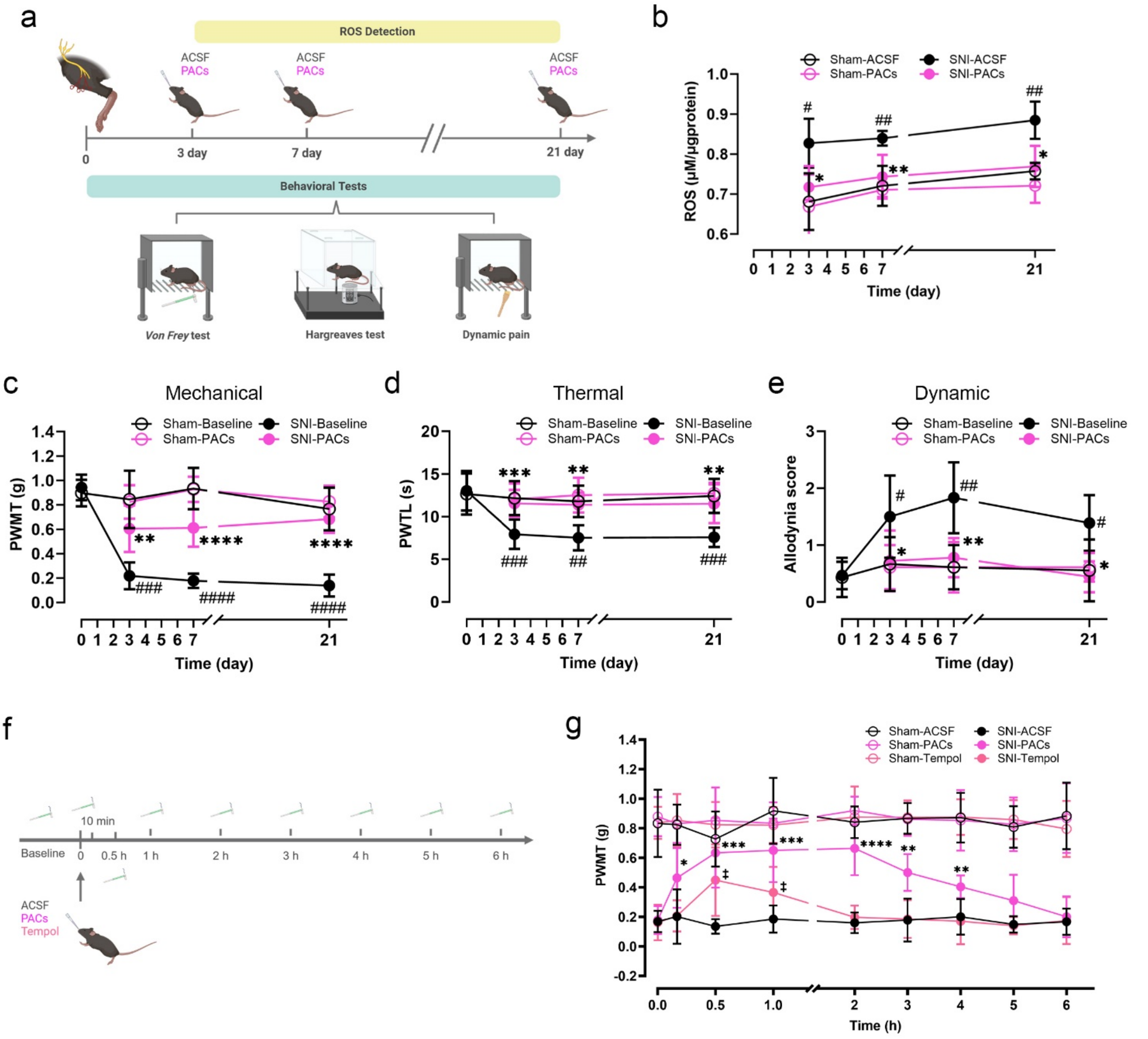

Supplementary Fig. 1. Temporal changes in pain behaviors, ROS levels, and PACs' analgesic effects. a, Experimental timeline and setup for ROS detection and pain behavioral tests. **b**, Quantification of ROS levels in the VLO over time, with measurements taken at day 3, 7, and 21 post-surgery in sham and SNI mice. **c-e**, Temporal changes in pain behaviors following SNI and PACs treatment: **c**, Mechanical pain sensitivity (PWMT) measured by *von Frey* test at days 3, 7, and 21 post-surgery; **d**, Thermal pain sensitivity (PWTL) measured by Hargreaves test at day 3, 7, and 21 post-surgery; **e**, Dynamic allodynia score measured by brushing the lesioned paw at day 3, 7, and 21 post-surgery. **f**, Timeline for assessing the duration of analgesic effects of PACs and Tempol at 7 days post-surgery using the *von Frey* test. **g**, Time course of PWMT measurements following

intranasal administration of PACs or Tempol, showing changes in pain threshold over a 6-hour period. Data are expressed as mean ± S.D. #: sham-ACSF *vs.* SNI-ACSF, *: SNI-PACs *vs.* SNI-ACSF, ‡: SNI-Tempol *vs.* SNI-ACSF $^{*/\#/‡}P < 0.05$, $^{**/\#\#}P < 0.01$, $^{***/\#\#\#}p < 0.001$, $^{****/\#\#\#\#}p < 0.0001$. See source data file for detailed statistical tests.

a

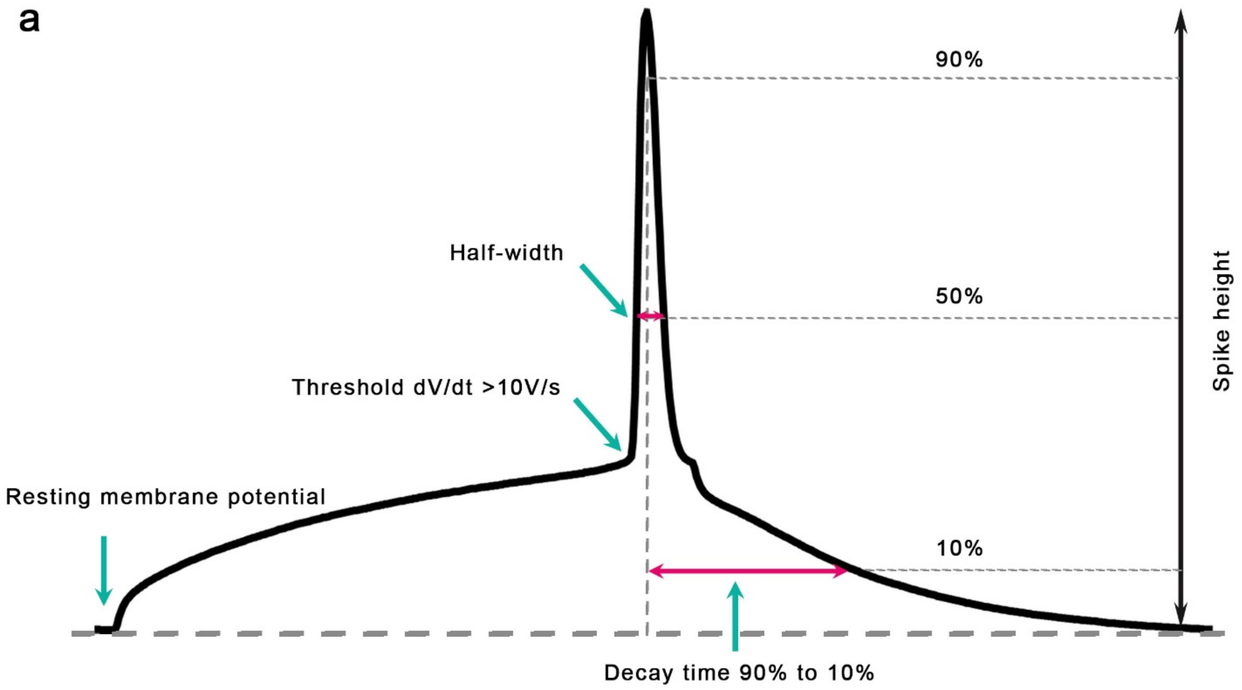

b

	Sham-Baseline	Sham-PACs	SNI-Baseline	SNI-PACs
AP threshold (mV)	-36.05 ± 0.5545	-36.93 ± 1.324	-39.58 ± 0.9157**	-37.97 ± 0.6133
Spike Height (mV)	114.1 ± 1.497	111.2 ± 1.674	114.3 ± 1.540	112.6 ± 1.658
Half- Width (ms)	1.861 ± 0.09948	1.920 ± 0.1380	1.535 ± 0.05326**	1.535 ± 0.05154
Rise time (ms)	0.2983 ± 0.01340	0.3156 ± 0.01702	0.2608 ± 0.005592*	0.2840 ± 0.009428
Max Rise slope (V/s)	188.5 ± 11.50	171.6 ± 13.43	209.7 ± 5.976	186.9 ± 7.300$^{##}$
Max Decay slope (V/s)	-59.34 ± 3.707	-59.26 ± 4.591	-74.22 ± 2.892**	-74.27 ± 2.373
Decay time 90%to 10% (ms)	22.23 ± 1.487	24.11 ± 2.237	17.89 ± 1.367*	22.03 ± 1.743$^{##}$

Supplementary Fig. 2. Summary of the electrophysiological parameters of AP of PYR^{VLO} in sham and SNI mice. a, A diagram displaying the electrophysiological parameters of AP elicited by rheobase current. **b**, Summary of the AP parameters of PYR^{VLO} in sham and SNI mice. Data are expressed as mean ± S.E.M. *: SNI-Baseline (n = 14) *vs.* Sham-Baseline (n = 14), two-tailed unpaired t test; #: SNI-PACs *vs.* SNI-Baseline, n = 14, two-tailed paired t test. $*/^{\#}P < 0.05$, $**/^{\#\#}P < 0.01$.

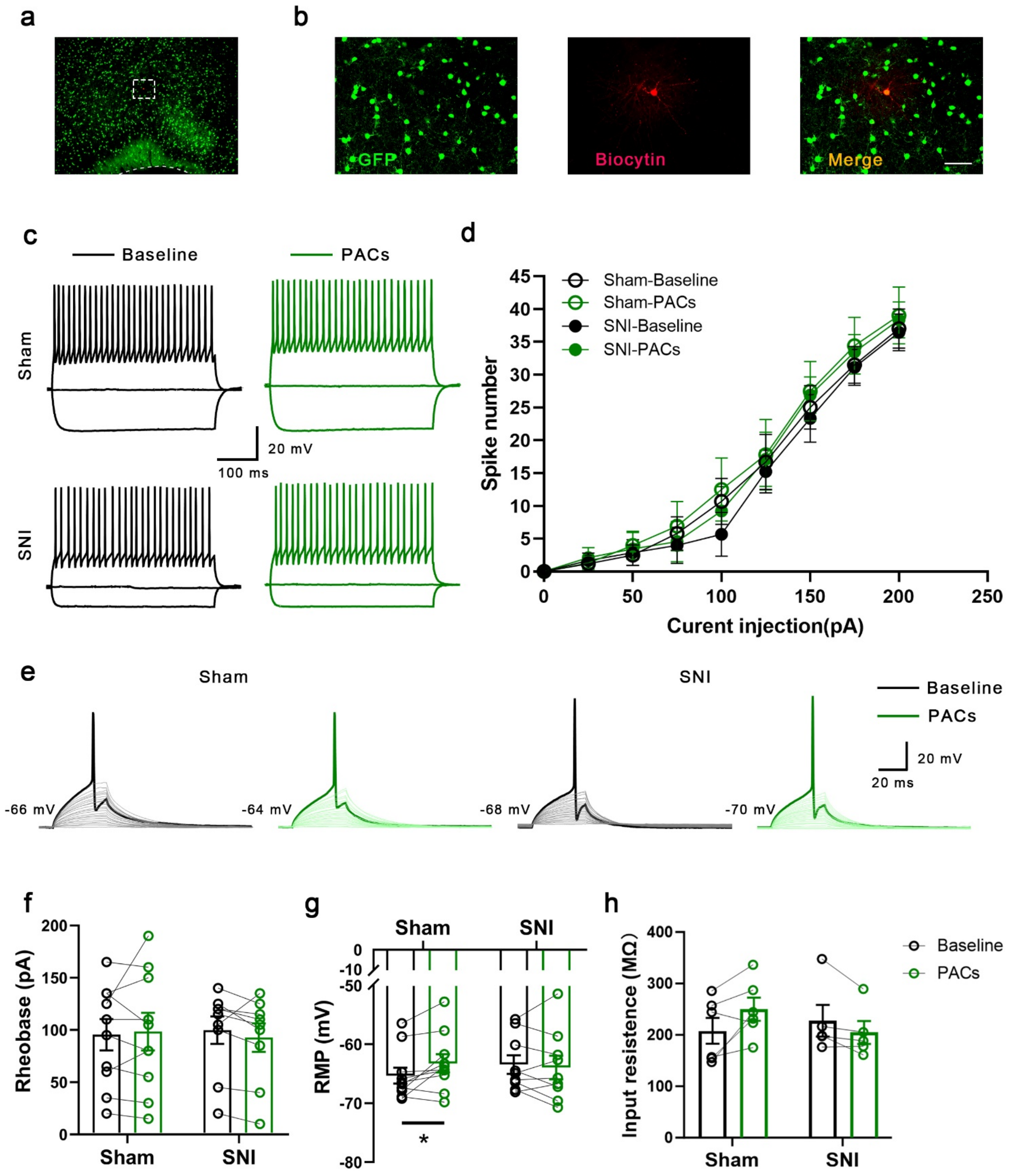

Supplementary Fig. 3. PACs have minimal impact on the activities of INT^{VLO}. **a**, GFP^{+} neurons in the VLO in a GAD67-GFP transgenic mouse. **b**, Images showing GFP^{+} interneurons (left), one whole-cell patched neuron labeled with biocytin and stained with TRITC (middle), and the merged image (right). **c**, Representative traces of action potentials elicited by 150 pA current stimulation before and during PACs application in sham

and SNI mice. **d**, Spikes evoked by step depolarizing currents before and during PACs application in sham (n = 10) and SNI (n = 9) mice. **e**, Representative traces of current threshold (rheobase) to elicit the first action potential before and during application of PACs in sham and SNI mice. **f**, Rheobase of INT^{VLO} before and during application of PACs in sham (n = 10) and SNI (n = 9) mice. **g**, RMP of INT^{VLO} before and during application of PACs in sham (n = 10) and SNI (n = 9) mice (Sham-Baseline *vs.* Sham-PACs, $p = 0.0268$). **h**, Input resistance of INT^{VLO} before and during application of PACs in sham (n = 6) and SNI (n = 5) mice (Sham-Baseline *vs.* Sham-PACs, $p = 0.0190$). Data are expressed as mean ± S.E.M. See source data file for detailed statistical tests. $^{*}p < 0.05$.

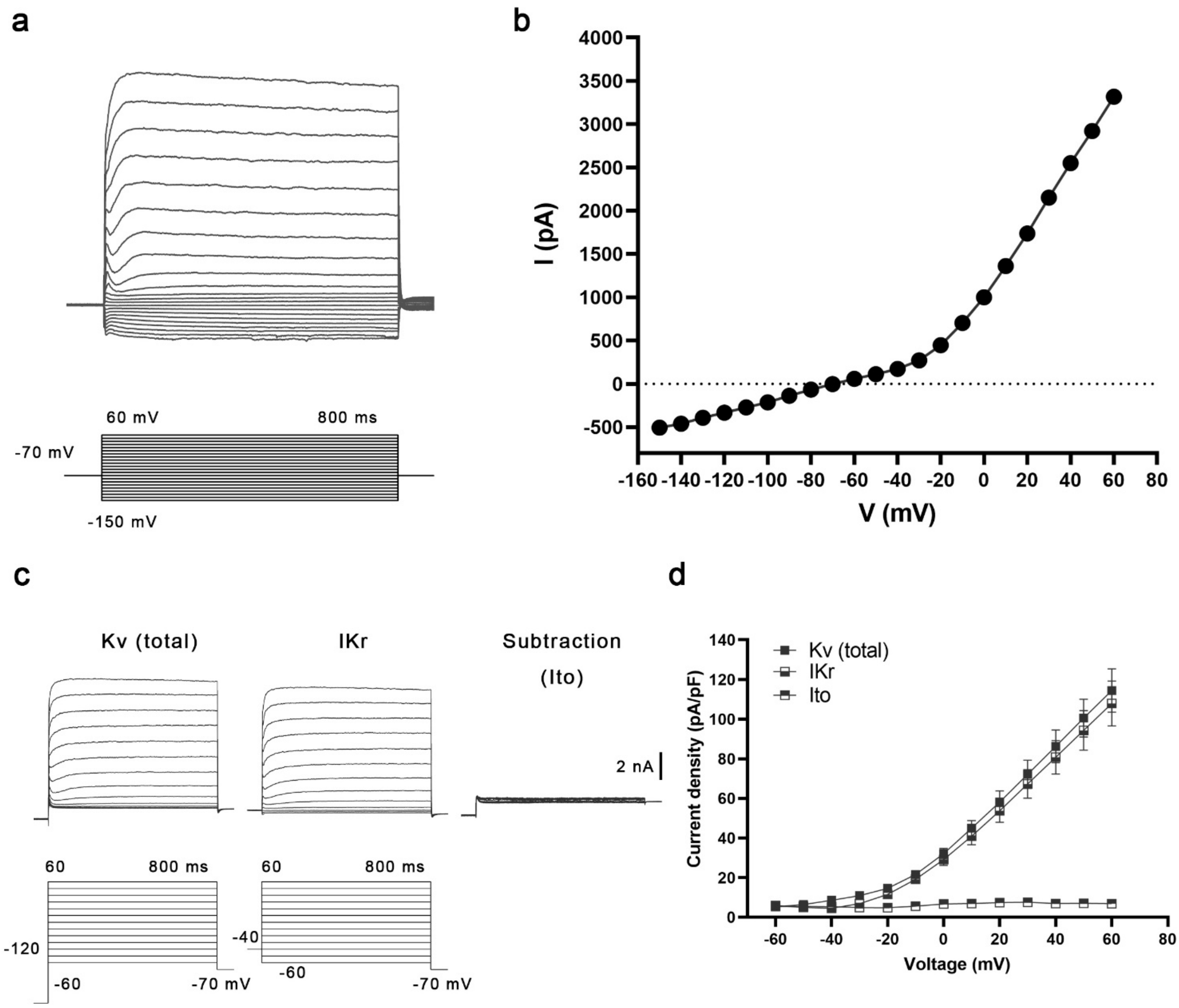

Supplementary Fig. 4. The delayed outward rectifier potassium current (Ikr) is the main component in the total voltage-dependent potassium (Kv) currents in PYR^{VLO}. **a**, Representative traces of total potassium currents (top) and the voltage command (bottom). **b**, I-V curve of total potassium currents for (a). **c**, Representative traces of total voltage-dependent potassium (Kv) current (left), delayed outward rectifier potassium (Ikr) current (middle), and transient outward potassium (Ito) current (right) obtained by subtraction Ikr from total Kv. **d**, Current density-voltage curves for total Kv, Ikr and Ito. Data are expressed as mean ± S.E.M. n = 8. See source data file for detailed statistical tests.

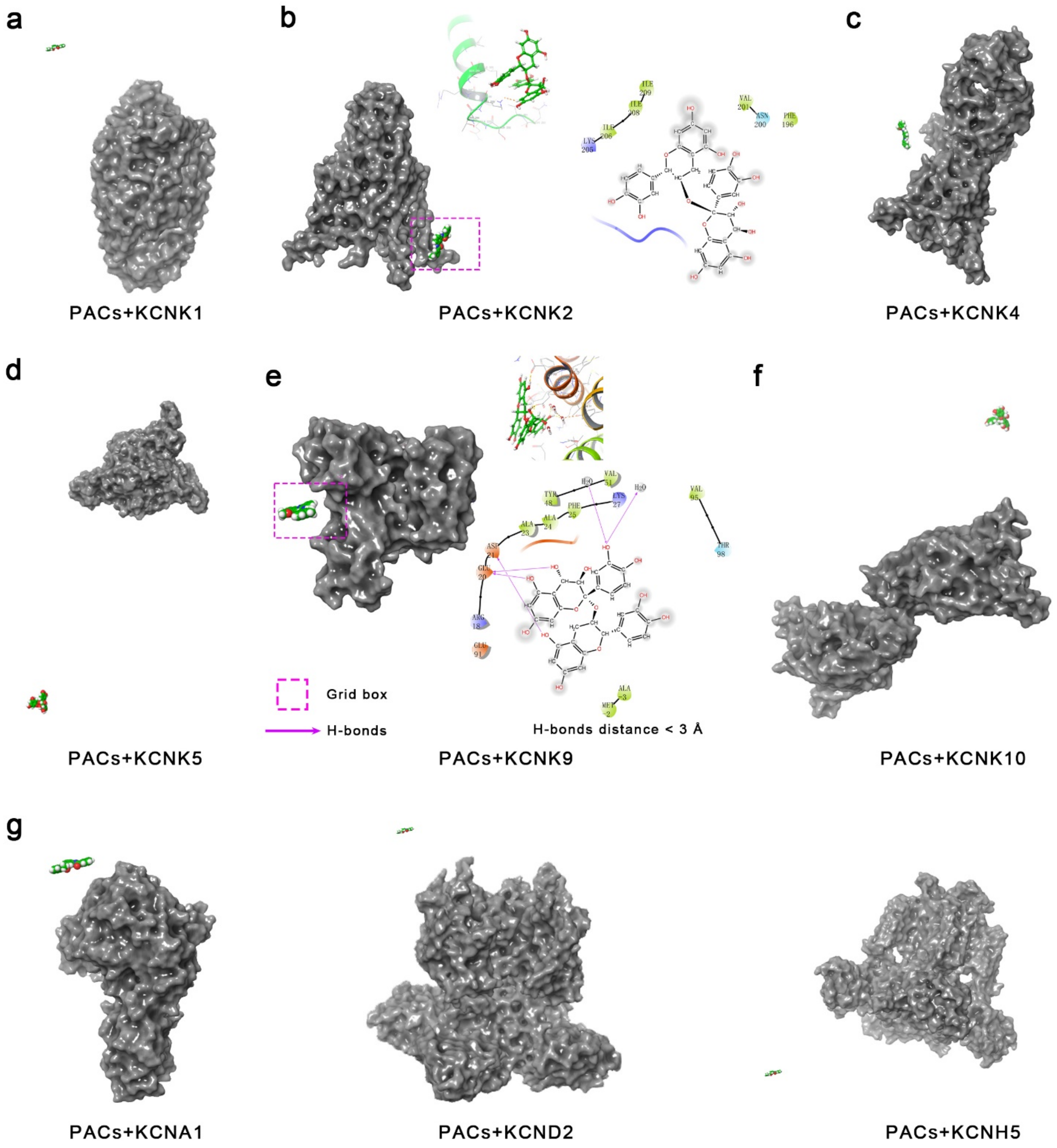

Supplementary Fig. 5. Prediction of PACs binding sites in other leak potassium channels and the PACs-downregulated Kv channels. a, PACs has no predicted binding site in KCNK1. **b**, The predicted binding site of PACs in KCNK2 is on the cytoplasmic domain. **c**, PACs has no predicted binding site in KCNK4. **d**, PACs has no predicted binding site in KCNK5. **e**, The predicted binding site of PACs in KCNK9 is on the transmembrane domain. **f**, PACs has no predicted binding site in KCNK10. **g**, PACs has no predicted binding sites in the

upregulated Kv channels in SNI mice.

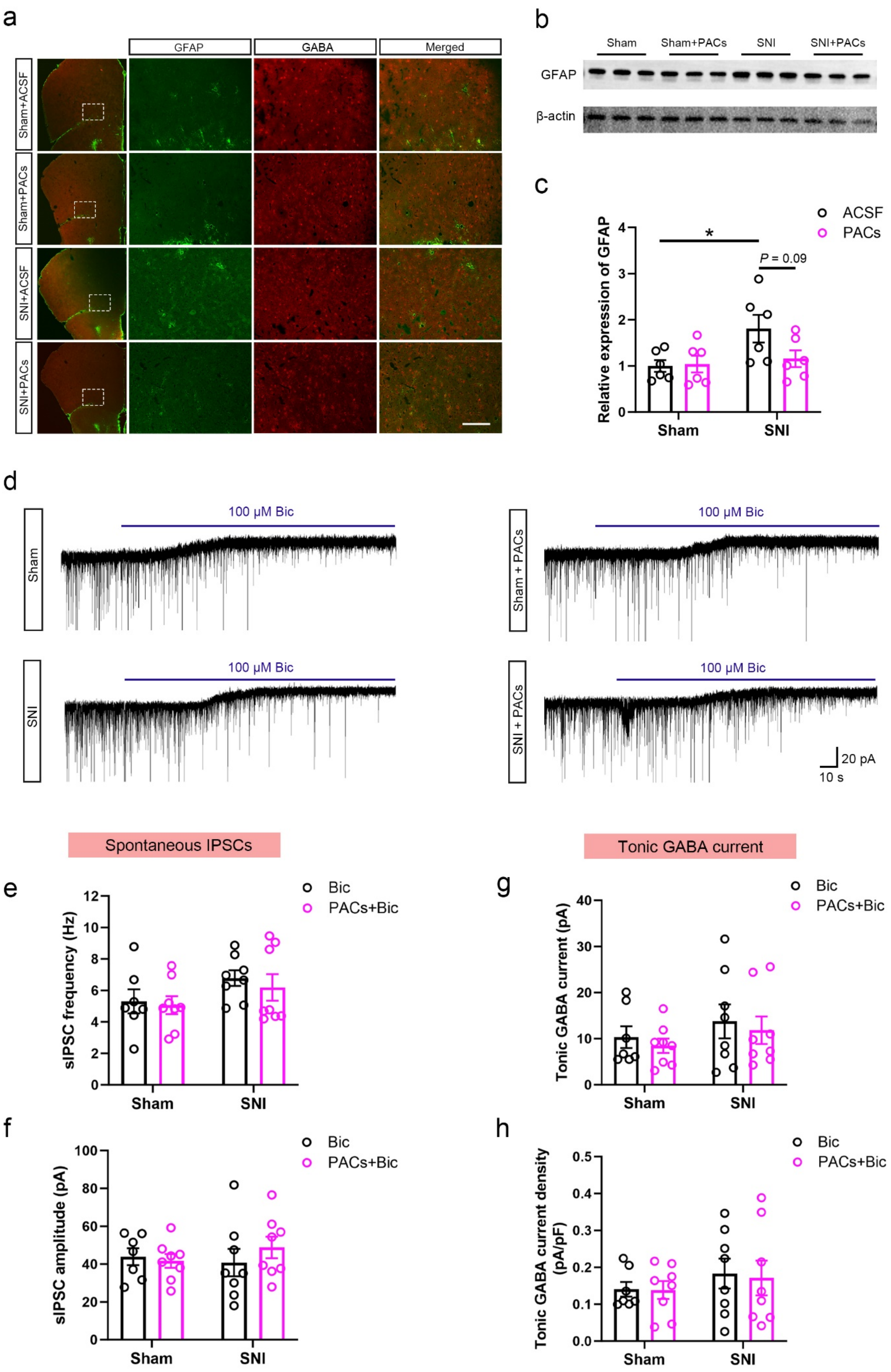

a
GFAP
GABA
Merged
Sham+ACSF
Sham+PACs
SNI+ACSF
SNI+PACs
b
Sham
Sham+PACs
SNI
SNI+PACs
GFAP
β-actin
c
ACSF
PACs
Relative expression of GFAP
P = 0.09
Sham
SNI
d
100 μM Bic
Sham
Sham + PACs
SNI
SNI + PACs
20 pA
10 s
Spontaneous IPSCs
Tonic GABA current
e
Bic
PACs+Bic
sIPSC frequency (Hz)
f
sIPSC amplitude (pA)
g
Tonic GABA current (pA)
h
Tonic GABA current density (pA/pF)

Supplementary Fig. 6. Astrocytes are activated after SNI, but PACs have no effect on the astrocytic GABA-mediated current. a, Immunofluorescence staining of GFAP (green) and GABA (red) in the VLO across different treatment groups. Scale bar = 100 µm. **b**, Western blot analysis of GFAP protein expression in the VLO across treatment groups, with β-Actin as the loading control. **c**, Summary data of relative GFAP expression levels: sham-ACSF (n = 6) vs. SNI-ACSF (n = 6), p = 0.0328. **d**, Representative traces of spontaneous IPSCs and tonic GABA-mediated current recorded in PYR^{VLO} in sham and SNI mice. **e-f**, summary data of frequency (**e**) and amplitude (**f**) of spontaneous IPSCs in Sham and SNI groups with and without PACs treatment. **g-h**, summary data of amplitude (**g**) and current density (**h**) of tonic GABAergic current in Sham and SNI groups with and without PACs treatment. Bic, bicuculline; sIPSC, spontaneous inhibitory postsynaptic currents. Data are expressed as mean ± S.E.M. *$P < 0.05$. See source data file for detailed statistical tests.
